# Physiological Changes and Time-Course Transcriptomic Analysis of Salt Stress in *Chenopodium quinoa*

**DOI:** 10.3390/biology14040416

**Published:** 2025-04-13

**Authors:** Peipei Li, Yemeng Zhang

**Affiliations:** College of Life Science and Bioengineering, Jining University, Qufu 273100, China; chamyemeng@163.com

**Keywords:** quinoa, salt stress, physiological, antioxidant enzyme, RNA-seq

## Abstract

Quinoa, a halophytic pseudocereal crop, is highly resistant to harsh growing environments and is considered a suitable crop for cultivation in marginal areas. The germination period plays a decisive role in the formation of the crop population and the growth and development of quinoa, but our understanding of the regulatory mechanism of salt stress remains limited. In this study, we investigated the physiological changes and mechanisms of tolerance response to salt stress in quinoa seedlings. The results showed that salt stress severely reduced the growth of quinoa seedlings. Moreover, salt stress increased the H_2_O_2_ level in the seedlings, thereby aggravating lipid peroxidation of the cell membrane and consequently increasing MDA content. Meanwhile, the antioxidant enzyme activities such as POD, SOD, GR and GPX of seedlings were enhanced in response to salt stress, which was consistent with the results of the RNA-sequencing. These results suggest that the increase in antioxidant enzyme activities in quinoa seedlings attenuates the ORS imbalance caused by salt stress. In addition, we identified 69, 40, 120 and 47 key genes in the “photosynthesis”, “glutathione metabolism”, “phenylpropanoid biosynthesis” and “starch and sucrose metabolism” pathways, respectively. Moreover, the predicted 235 transcription factors involved in the salt stress response have various hormone cis-elements in their promoter regions, which also indicates that multiple hormones are involved in the salt stress response process in quinoa. Therefore, we hope that these genes and mechanisms will provide some basis for understanding salt tolerance in quinoa.

## Simple Summary

In this study, we observed growth and physiological changes in quinoa (*Chenopodium quinoa* Willd.) seedlings under salt stress. We found that quinoa reduced ROS imbalance and cell membrane damage caused by salt stress mainly by enhancing antioxidant capacity, which was, in general, consistency with the results of time-course RNA-sequencing. In addition, we also identified transcription factors and key genes in various pathways, such as “photosynthesis”, “glutathione metabolism”, “phenylpropanoid biosynthesis” and “starch and sucrose metabolism”, by transcriptomic data. This study provides an important theoretical basis for the subsequent genetic improvement of this species, as well as a potential candidate for the remediation of saline-alkali soils.

## 1. Introduction

Salinization is one of the most serious abiotic threats to global agricultural productivity and ecosystems, with approximately half of the world’s irrigated land threatened by salinity [1,2]. A series of metabolic disorders caused by soil salinization, such as ionic toxicity, drought stress and oxidative stress, significantly reduce the yield and quality of crops [3]. Environmental change and population growth have further exacerbated the extent and size of soil salinization, forcing agricultural production to expand to marginal areas [4]. Hence, improving the salinity tolerance of crops and rehabilitating saline land represent agricultural production problems that must be urgently solved. Long-term amelioration practices reshaping the soil suggest that saline agriculture can make use of saline soil and brackish water resources to directly plant halophytic or salinity-tolerant crop varieties on saline-alkali land, which can both promote the improvement of saline-alkali land and additionally increase agricultural ecological diversity and agricultural production [5,6]. However, most traditional crops and forages are glycophytes, which are highly sensitive to salt stress [7]. Therefore, the use of halophytic crops with high salinity tolerance is a viable option [8].

*Chenopodium quinoa* Willd., a crop in the family Amaranthaceae, is an ancient crop native to South America. Because quinoa is highly resistant to multiple abiotic stresses and its seed has better nutritional value than other major cereals, quinoa has attracted worldwide attention [9,10]. Quinoa is a halophytic pseudocereal with a high salt tolerance and can cope with high salt stress (conductivity 40 dS·m^−1^) [11]. Na^+^ segregation in vesicles, K^+^ retention [12], xylem Na^+^ loading [13], higher ROS tolerance and effective regulation of stomatal development and size [14] are key to the adaptation and survival of quinoa in saline-alkali environments. In addition, the accumulation of compatible solutes, such as proline, total phenolics, Betalains, choline and polyamines, is associated with salt tolerance in quinoa [15]. Despite a large number of studies on the mechanisms of salt response and tolerance in quinoa, there remain relatively few studies at the transcriptional level during the germination period. Compared with glycophyte plants, some halophytes are also relatively sensitive to salt during the germination and seedling periods, whereas quinoa has higher resistance to high salt stress during germination [16]. Hariadi et al. found that NaCl concentrations higher than 400 mM have a significant inhibitory effect on seed germination and concluded that seed germination and viability depend on the ability to excrete toxic Na^+^ from developing embryos, thereby avoiding ionic toxicity [12]. However, Burrieza et al. suggested that the high tolerance of quinoa germination is due to the significant gradient distribution of toxic ions (Na^+^ and Cl^−^) and necessary ions (K^+^, Mg^2+^, Ca^2+^, etc.) in the seed coat [17]. Moreover, sufficient evidence has demonstrated that salt stress can lead to the excessive production of ROS in quinoa, such as hydrogen peroxide (H_2_O_2_) and superoxide radicals (O^2−^). These ROS can cause serious damage to proteins, DNA, lipids and chlorophyll in plant cells. However, the higher antioxidant enzyme activity can eliminate excess ROS in quinoa. Hassan et al. showed a strong positive correlation between antioxidant enzymes and inorganic ions, and that elevated antioxidant enzyme activity has the potential to mitigate salt stress injuries by promoting the absorption of inorganic solutes for osmoregulation in quinoa plants [18]. Thus, the molecular basis of the germination period, as a key process in quinoa life history and the formation of crop field populations, is worth further exploration.

Recently, the completion of the quinoa genome and the development of high-throughput sequencing have provided new approaches to address complex salt stress-related pathways [19,20]. Schmöckel et al. integrated RNA-sequencing and SNP analysis to identify 219 candidate genes associated with salt stress, predicting the transmembrane structure of these genes as well as demonstrating specificity or overexpression in quinoa compared with other Amaranthaceae [21]. In addition, a comparison of epidermal salt bladder and leaf lamina cells in quinoa revealed that quinoa stress resistance was related to ion and nutrient transport, ABA homeostasis and signaling, enhanced basal-level ABA responses, and other pathways [8]. To this end, Qingli No. 4 (approved by Qinghai, China, 2019001) was used as the material to analyze the changes in the transcription level of seedlings under 300 mM NaCl treatment to understand the salt tolerance mechanism. Differentially expressed genes (DEGs) at different treatment times were obtained using RNA-seq. Potential positive and negative regulatory factors under salt stress conditions were screened by KEGG enrichment analysis and TF prediction. Therefore, the results of this study could enhance the knowledge of salt stress regulation in quinoa, with a view to providing a resource of target gene data for performing multiomics cooperation.

## 2. Materials and Methods

### 2.1. Plant Material

Qingli No. 4, an alpine ecotype variety that is approved by Qinghai Province (accession number: 2019001), were propagated and preserved by the Qinghai Provincial Key Laboratory of Crop Molecular Breeding. Approximately 300 seeds were presterilized in 70% (*v*/*v*) ethanol/water for 5 min and then subjected to several rinses in ddH_2_O. Fifty seeds per box were placed in a seed germination box with two layers of filter paper. Twelve milliliters of distilled water (control) and 100, 200, 300, 400 and 500 mM NaCl were added to each box and placed in a 12 h (25 °C) light/12 h (20 °C) dark artificial climate incubator for germination. The germination rate was calculated by three replicates of each treatment. Those with 1 mm long rootlets were recognized as germinated seeds.

After 1 d of seed germination under normal conditions, seedlings in good and uniform growth status were selected for 300 mM NaCl stress treatment, and whole plants were sampled at 0, 24, 48 and 72 h, frozen rapidly in liquid nitrogen and stored at −80 °C for subsequent RNA-seq.

### 2.2. Measurement of Malondialdehyde Levels

The malondialdehyde (MDA) content of the quinoa experimental group and control group was measured using thiobarbituric acid (TBA, purchased from Sangon Biotech, Shanghai, China), as previously reported. Samples (0.5 g) were ground in 2.5 mL of reagent (0.25% (*w*/*v*) TBA in 10% (*w*/*v*) trichloroacetic acid) and boiled at 100 °C for 20 min. The MDA content was determined by subtracting the nonspecific absorption at 600 nm from the absorbance of the sample supernatant at 532 nm.

### 2.3. Measurement of H_2_O_2_ Levels and Antioxidant Enzyme Activities

The extraction procedures for antioxidant enzymes and H_2_O_2_ were performed at 4 °C. Samples of quinoa seedings (0.5 g) were crushed and mixed in 2 mL of extraction buffer (0.1 M potassium phosphate buffer with 0.1 mM EDTA (purchased from Sangon Biotech, Shanghai, China), pH 7.0) and centrifuged at 15,000× *g* in a refrigerated centrifuge for 20 min. H_2_O_2_ content was determined according to the method described by Jena and Choudhuri [22]. A 3 mL sample of supernatant was mixed with 1 mL of 0.1% TiCl_4_ in 20% H_2_SO_4_ (*v*/*v*), and the mixture was then centrifuged at 6000× *g* for 15 min at room temperature. The optical absorption of the supernatant was measured spectrophotometrically at 410 nm to determine the H_2_O_2_ content. The activities of SOD, POD, GR and GPX were measured according to the NBT photochemical reduction method [23], Guaiacol method [24], UV-absorption method [25] and DTNB method [22], respectively, and the assays were carried out with an assay kit (purchased from Comin, Suzhou, China). The procedures were performed as described by the Suzhou Comin Biotechnology Research Institute.

### 2.4. RNA-Seq and Data Processing

Total RNA from each sample was extracted using TRIzol reagent (Gibco, Grand Island, NY, USA) according to the manufacturer’s instructions. DNA was removed using RNase-free DNase I (Takara, Dalian, China). The purity, concentration and integrity of each total RNA sample were carefully measured by a NanoDrop spectrophotometer (Thermo Scientific-1000, Thermo Fisher Scientific, Wilmington, DE, USA), and only RNA integrity numbers higher than 8.0 were used for downstream sequencing library construction. First-strand cDNA was synthesized using M-MLV reverse transcriptase (Sangon, Shanghai, China). RNA-seq was then performed on the Illumina (HiSeq TM) platform at Sangon, Shanghai, China. Data quality control of raw reads was performed using Trimmomatic to obtain clean reads. Clean reads were compared to the quinoa reference genome (https://plants.ensembl.org/Chenopodium_quinoa/Info/Index accessed on 6 January 2025) using HISAT2 (v2.2.1) software [26]. To estimate the gene expression levels, the transcripts per million fragments mapped (TPM) value of each gene was calculated. DEseq2 (v1.36.0) [27] was used to screen for DEGs. The threshold was *p* < 0.05 and fold change |(FC)| > 2.

### 2.5. K-Means Clustering and KEGG Enrichment Analysis of DEGs

We performed k-means clustering analysis of all DEGs using the k-means function in R (v3.2.2). Heatmaps were drawn using the Pheatmap package in R and were clustered using Pearson correlation distance. The KEGG enrichment analysis of the DEGs was implemented using KOBAS (v3.0) software [28]. Pathways with correlated *p* values less than 0.05 were assigned as significantly enriched items.

### 2.6. Identification of Transcription Factors from DEGs and Discovery of Cis-Regulatory Elements in the Promoter Regions

TFs were identified using the online forecasting tool Plant Transcription Factor Database v4.0 (http://planttfdb.cbi.pku.edu.cn accessed on 20 January 2025) [29]. With default parameters for TF family assignment and thresholds, the TFs were identified out of DEGs. Afterward, the promoter sequences (approximately 2.0 kbps upstream of the transcription start site) of identified TFs were downloaded from the quinoa reference genome database (https://plants.ensembl.org/Chenopodium_quinoa/Info/Index accessed on 6 January 2025). Cis-element prediction was performed using the online forecasting tool Plant CARE (http://bioinformatics.psb.ugent.be/webtools/plantcare/html/ accessed on 16 February 2025) [30]. The promoter region was enriched by the online forecasting tool MEME (https://meme-suite.org/meme/ accessed on 16 February 2025), and the identified DNA motifs were aligned with the motif database to obtain the best matched known regulatory motifs in Arabidopsis by TOMTOM [31].

### 2.7. Real-Time Quantitative Reverse Transcription-Polymerase Chain Reaction (RT–qPCR)

Total RNA was extracted from the control and salt-treated samples collected at 24, 48 and 72 h and used to quantify gene expression in each group. Actin (*ACT-1*) was used as the reference gene to normalize and estimate up- or down-regulation of the target genes. Transcript levels are shown as the mean normalized expression (MNE). All primers are listed in the Supplementary Materials (Table S1 and Figure S1). Three biological replicates were performed per group.

### 2.8. Statistical Analysis

Statistical analysis was performed using SPSS (v25.0) software. Variances in germination rates under varying salt concentrations were analyzed using a one-way ANOVA followed by an LSD test. Physiological traits under varying salt time points were analyzed using an independent sample *t*-test. Significance levels were set at *p* < 0.05.

## 3. Results

### 3.1. Seed Germination Assay

**Figure 1:**
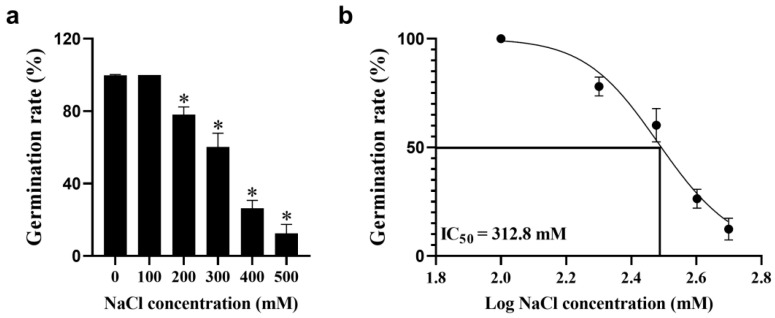
Germination test of quinoa seeds under CK and NaCl treatment. Germination rate under CK and varying NaCl concentrations. (**a**). Significant differences in germination rate among varying NaCl concentrations (**b**). The half maximal inhibitory concentration of quinoa seed germination under varying NaCl concentrations. Data are presented as mean ± SD, n = 3. * *p* < 0.05.

To test the tolerance of quinoa seeds to salt stress, the germination rates of seeds treated with distilled water (CK) and 100, 200, 300, 400 and 500 mM NaCl were calculated. The results indicated that 100 mM NaCl had no significant effect on seed germination, but the germination rate of quinoa seeds gradually decreased with increasing salt concentration (Figure 1a), and salinity significantly inhibited the growth of radicles and germ cells (Figure S2). In addition, the half maximal inhibitory concentration (IC_50_) of NaCl on quinoa seed germination was 312.8 mM (Figure 1b). Therefore, it is speculated that quinoa also has a high tolerance to salt stress during the germination period.

### 3.2. Cell Membrane Damage and H_2_O_2_ Production Under CK and NaCl Stress

**Figure 2:**
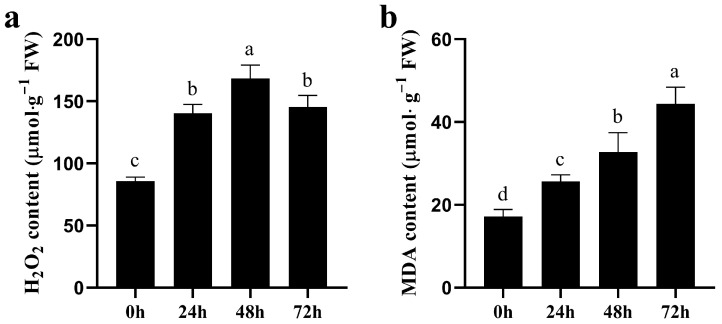
Content of H_2_O_2_ and MDA in quinoa under CK and NaCl treatment. H_2_O_2_ content in quinoa seedlings under varying NaCl treatment times (**a**). MDA content in quinoa seedlings under varying NaCl treatment times (**b**). Significant differences in H_2_O_2_ and MDA content among varying NaCl treatment times are represented by distinct lowercase.

Generally, salt stress leads to an imbalance in ROS content in plant cells, causing damage to the cell membrane. Therefore, we measured the MDA and H_2_O_2_ content of quinoa seedings. In quinoa seedlings, as the duration of salt stress increased, the H_2_O_2_ content increased and then decreased (Figure 2a), whereas the MDA content increased (Figure 2b). This relationship demonstrates that salt stress disrupts normal physiological processes and causes damage to the membrane system in quinoa.

### 3.3. Activities of Antioxidant Enzymes in Quinoa Under CK and NaCl Stress

**Figure 3:**
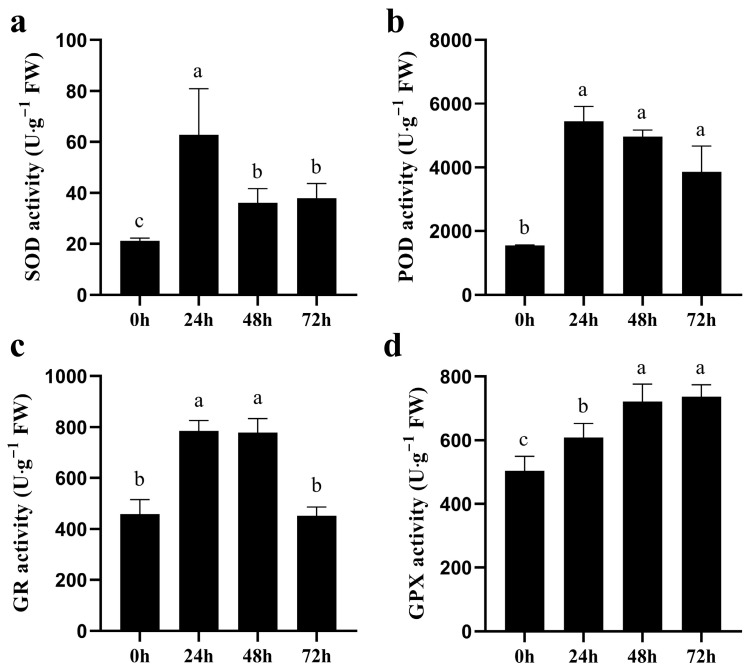
Activities of antioxidant enzymes in quinoa under CK and NaCl treatment. SOD (**a**), POD (**b**), GR (**c**) and GPX (**d**) activities in quinoa seedlings under varying NaCl treatment times. Significant differences in activities of antioxidant enzymes among varying NaCl treatment times are represented by distinct lowercase.

Normally, when ROS are imbalanced in plants, the activity of antioxidant enzymes is altered accordingly. In quinoa seedlings, as the duration of salt stress increased, the SOD, POD and GR activities increased and then decreased, whereas the GPX activity increased (Figure 3). This result suggests that excess ROS caused by salt stress can be eliminated by increasing the activity of antioxidant enzymes during quinoa germination.

### 3.4. Time-Course Transcriptional Profiles of Seedlings in Quinoa

**Figure 4:**
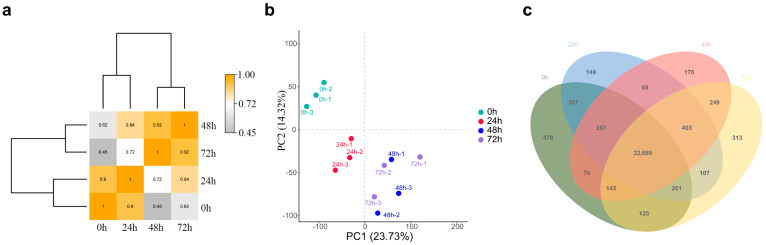
Gene expression analysis between samples. Cluster analysis (**a**), PCA analysis (**b**) and Venn analysis (**c**) between tgroups.

To explore response genes at the seedling stage under salt stress, quinoa seedlings were treated with 300 mM NaCl (~IC_50_) 1 day after germination as the stress treatment and then sampled at 0, 24, 48 and 72 h for RNA-seq analysis. The 12 RNA-seq samples generated approximately 69~102 hundred million bases, and more than 90% of the input reads could be compared to the quinoa reference genome (Table 1). A threshold of the mean TPM > one of genes in the three replicates was used to identify the expressed genes. We found that the number of expressed genes at 24 h (24,251), 48 h (24,075) and 72 h (24,292) after salt stress was altered compared to that at 0 h (24,350) (Table 2). According to the gene expression level, group clustering found that the 0 and 24 h samples were clustered as adjacent clades, distant from the 48 and 72 h clades, indicating that the expression patterns of many genes changed with increasing stress time (Figure 4a). In addition, the PCA of the different sample transcript profiles could be divided into three groups: 0 h, 24 h, and 48 and 72 h (Figure 4b), which is consistent with the results of cluster analysis. The Venn diagram indicated that 22,689 genes were commonly expressed in all samples, with 478, 149, 176 and 313 genes specifically expressed at 0, 24, 48 and 72 h, respectively (Figure 4c). This result suggests that salt stress represses the expression of a large number of genes, but as the duration of salt stress increases, some genes involved in the regulatory mechanisms of salt stress are activated. It is inferred from the results of PCA and cluster analysis that the early (24 h) involvement of pathways or genes in salt stress regulation may be different from the later stage (48 and 72 h).

### 3.5. KEGG Enrichment Analysis Between Groups

**Figure 5:**
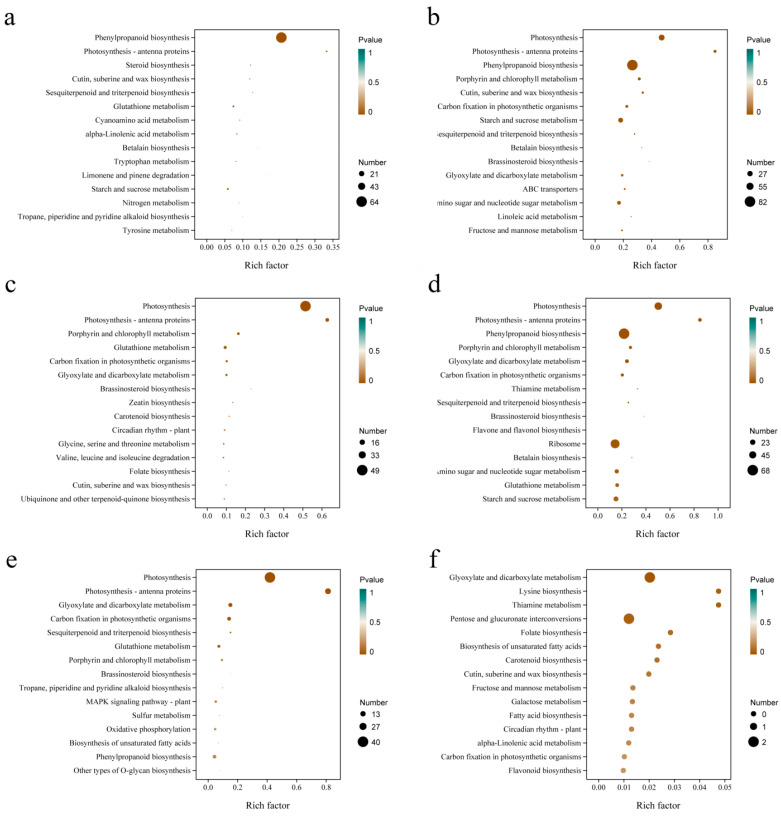
KEGG enrichment analysis of the DEGs at 24 h (**a**), 48 h (**b**), 48 h vs. 24 h (**c**), 72 h (**d**), 72 h vs. 24 h (**e**), and 72 h vs. 48 h (**f**).

A total of 5486 DEGs with *p* values < 0.05 and fold change |(FC)| > 2 were screened by comparison between groups (Table 3). There were 1078 DEGs at 24 h under salt stress, which was much less than the number of DEGs at 48 (3145) and 72 h (3112), but only 76 DEGs were consistent between 48 and 72 h, which further indicated that there were differences in the regulatory mechanism of quinoa in the early and late stages of the salt stress response. KEGG enrichment analysis revealed that DEGs at 24 h under salt stress were highly enriched in “phenylpropanoid biosynthesis” (Figure 5a). The 48 h samples were primarily enriched in “photosynthesis”, “phenylpropanoid biosynthesis” and “starch and sucrose metabolism” (Figure 5b), whilst the 72 h samples were primarily enriched in “photosynthesis”, “phenylpropanoid biosynthesis” and “ribosome” (Figure 5d). KEGG enrichment analysis of DEGs at 48 h vs. 24 h, 72 h vs. 24 h and 72 h vs. 48 h indicated that 48 and 72 h vs. 24 h were primarily enriched in “photosynthesis” and “photosynthesis-antenna proteins” (Figure 5c,e). The comparison of 72 h vs. 48 h identified enrichment primarily in “glyoxylate and dicarboxylate metabolism” and “pentose and glucuronate interconversions” (Figure 5f). Thus, there are not only differences in the number of DEGs in the early (24 h) and late (48 and 72 h) stages of salt stress, but also in the regulatory mechanisms involved.

### 3.6. K-Means Clustering of DGEs

**Figure 6:**
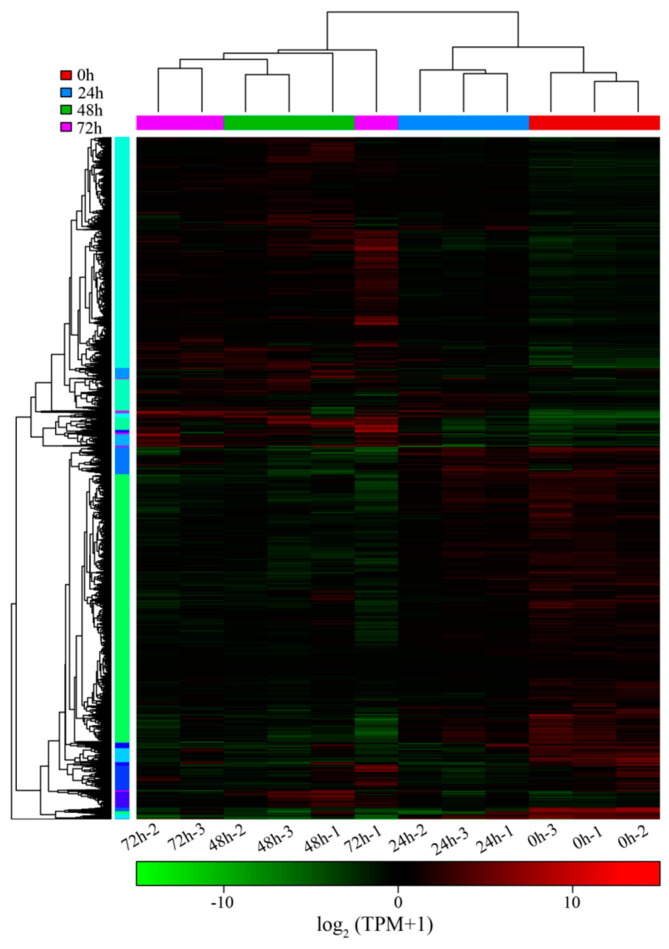
K−means cluster analysis of the DEGs.

**Figure 7:**
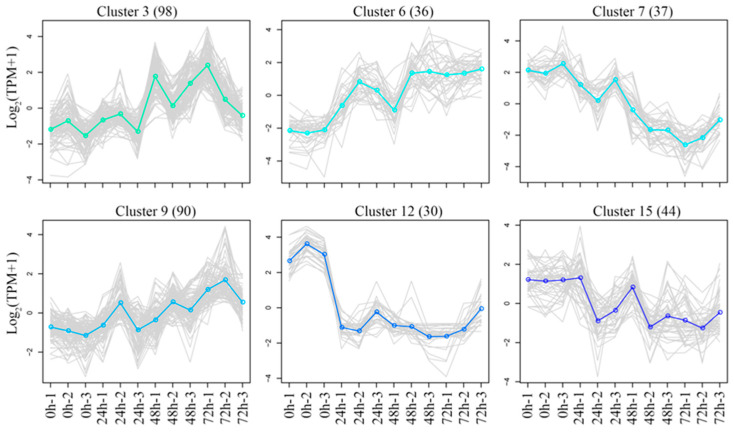
Obvious expression patterns of genes in the 30 k−means cluster.

We performed a k-means clustering analysis of the 5165 DEGs using the k-means function in R (Figure 6). A total of 30 clusters with various gene expression dynamics were identified. Significantly, Clusters 3, 6, 7, 9, 12 and 15 exhibited obviously specific expression patterns (Figure 7). The transcription levels of 98, 36 and 90 genes contained in Clusters 3, 6 and 9, respectively, increased with increasing stress time, whereas the transcription levels of 37, 30 and 44 genes contained in Clusters 7, 12 and 15, respectively, decreased with increasing stress time. KEGG analysis of six clusters with significant specific expression patterns revealed that Cluster 3 was significantly enriched in “phenylpropanoid biosynthesis”, “zeatin biosynthesis” and “glutathione metabolism”, Cluster 6 was enriched in “phenylpropanoid biosynthesis”, Cluster 7 was enriched in “photosynthesis-antenna proteins”, Cluster 9 was enriched in “phenylpropanoid biosynthesis” and “cysteine and methionine metabolism”, Cluster 12 was enriched in “phenylpropanoid biosynthesis”, and Cluster 15 was enriched in “phenylpropanoid biosynthesis”, “ubiquitin mediated proteolysis”, “RNA polymerase” and “protein processing in endoplasmic reticulum” (Table 4). These results suggest that quinoa coping strategies for salt stress are multifaceted and complex, and DEG enrichment pathways can provide important clues for the regulatory mechanism of quinoa salt stress.

### 3.7. KEGG Enrichment Analysis of DEGs Under Salt Stress

**Figure 8:**
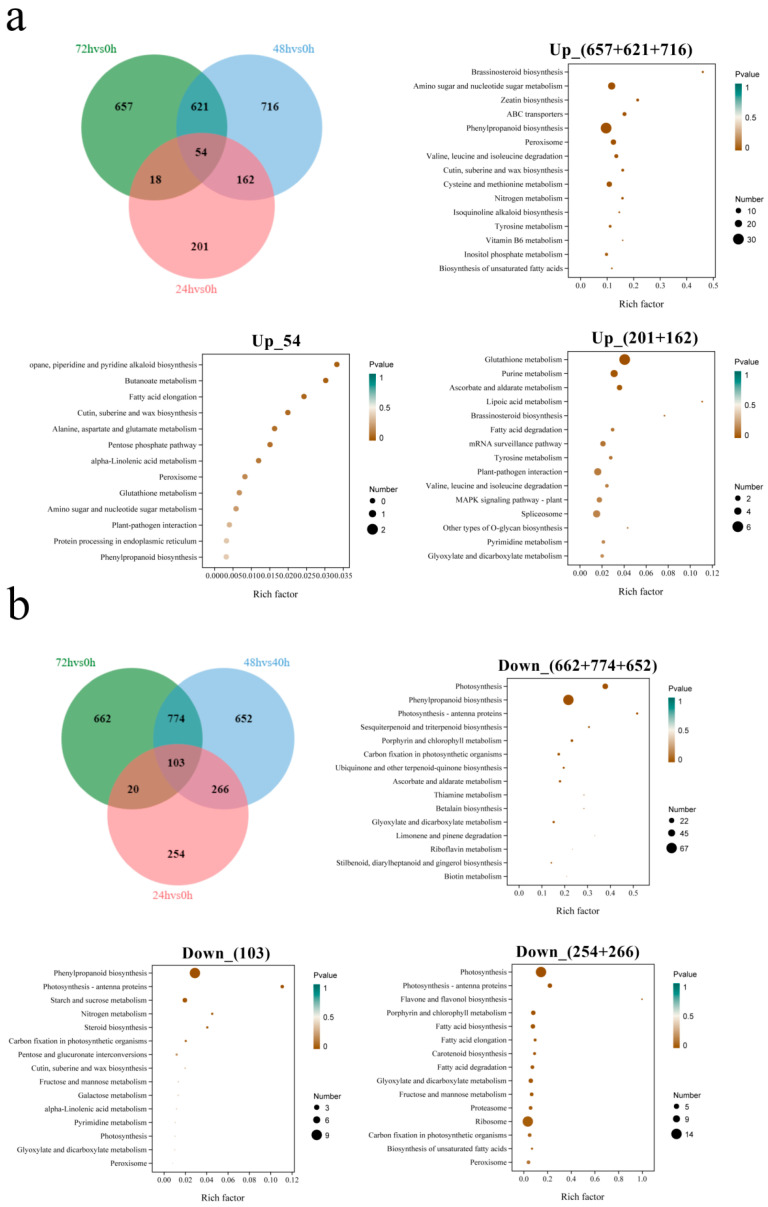
Venn diagram and KEGG enrichment analysis of the up-regulated (**a**) and down-regulated (**b**) genes between comparisons of 72 h vs. 0 h, 48 h vs. 0 h and 24 h vs. 0 h.

Salt stress has an impact on the entire growth and development process of quinoa; not only will early osmotic stress lead to the loss of quinoa water, but later ionic toxicity will also lead to an imbalance in the ROS [32]. Therefore, to explore genes that are highly correlated with the regulatory mechanisms of salt stress, we compared the DEGs of 72 h vs. 0 h, 48 h vs. 0 h and 24 h vs. 0 h. As shown in Figure 6, there were 1395 DEGs co-expressed in 72 h vs. 0 h and 48 h vs. 0 h, 1319 DEGs specifically expressed in 72 h vs. 0 h and 1368 DEGs specifically expressed in 48 h vs. 0 h. KEGG enrichment revealed that these up-regulated DEGs were enriched in “phenylpropanoid biosynthesis” (Figure 8a) and down-regulated in “phenylpropanoid biosynthesis” and “photosynthesis” (Figure 8b). We speculate that these pathways may be involved in the regulatory mechanisms of quinoa under salt stress in later stages. A total of 157 DEGs were co-expressed in 72 h vs. 0 h, 48 h vs. 0 h and 24 h vs. 0 h. KEGG enrichment indicated that there was no significant enrichment pathway for these up-regulated DEGs (Figure 8a). The down-regulated DEGs were primarily enriched in “phenylpropanoid biosynthesis” and “starch and sucrose metabolism” (Figure 8b). However, 455 DEGs were specifically expressed in 24 h vs. 0 h, and 428 DEGs were co-expressed in 48 h vs. 0 h and 24 h vs. 0 h. KEGG enrichment indicated that these up-regulated DEGs were enriched in “glutathione metabolism” (Figure 8a) and down-regulated in “photosynthesis” and “ribosome” (Figure 8b). We speculate that these pathways may be involved in the regulatory mechanisms of quinoa under salt stress in the early stage. Since the co-expressed DEGs of 72 h vs. 0 h and 24 h vs. 0 h are unlikely to participate in salt stress regulation, no further analysis was performed.

### 3.8. Salt Stress Treatment Affected the Expression of Photosynthetic Pathway Genes in Quinoa Seedlings

**Figure 9:**
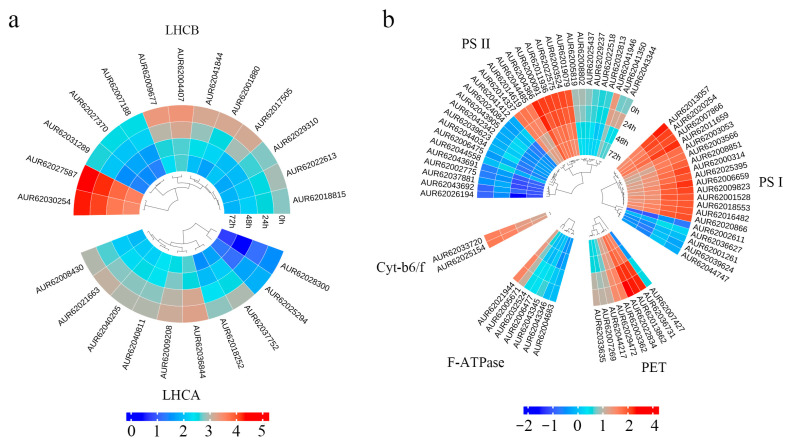
Heatmap of expression patterns of DEGs in photosynthesis (**a**) and photosynthesis-antenna (**b**).

Through KEGG enrichment analysis, we found that salt stress had a greater effect on the photosynthesis of quinoa seedlings. The “photosynthesis” and “photosynthesis-antenna proteins” pathway mapping of DEGs revealed that a total of 92 key genes for photosynthesis were regulated by salt stress. Ten *LHCA* and thirteen *LHCB* photosynthetic antennatin genes were found in the “photosynthesis-antenna proteins” pathway (Figure 9a). In addition, there were 69 DEGs in the “photosynthesis” pathway, of which twenty Psas6 genes were involved in PS I, thirty-one *Psb* genes in PS II, nine *Pet* genes in photosynthetic electron transport (PET) and seven affecting F-ATPase activity. Of note is the discovery of two Cyt-b6/f genes (Figure 9b). The expression levels of these genes were down-regulated to varying degrees under salt stress. The KEGG map revealed that the photosynthesis of quinoa seedlings was not significantly reduced at 24 h, and only two PsaL genes involved in the PS I process were down-regulated. However, at 24–48 h, all stages of photosynthesis were severely affected, but photosynthesis did not decreased between 48–72 h. The “photosynthesis-antenna protein” pathway has a similar time dependence to “photosynthesis”. This indicates that when quinoa seedlings are exposed to salt stress, photosynthesis does not decline immediately and will not change until a threshold is reached. Quinoa, as a halophytic pseudocereal plant, is tolerant to salt stress, but chronic stress can also lead to an imbalance in its physiological processes.

### 3.9. Salt Stress Treatment Affects the Expression of of Glutathione Metabolism Genes in Quinoa Seedlings

**Figure 10:**
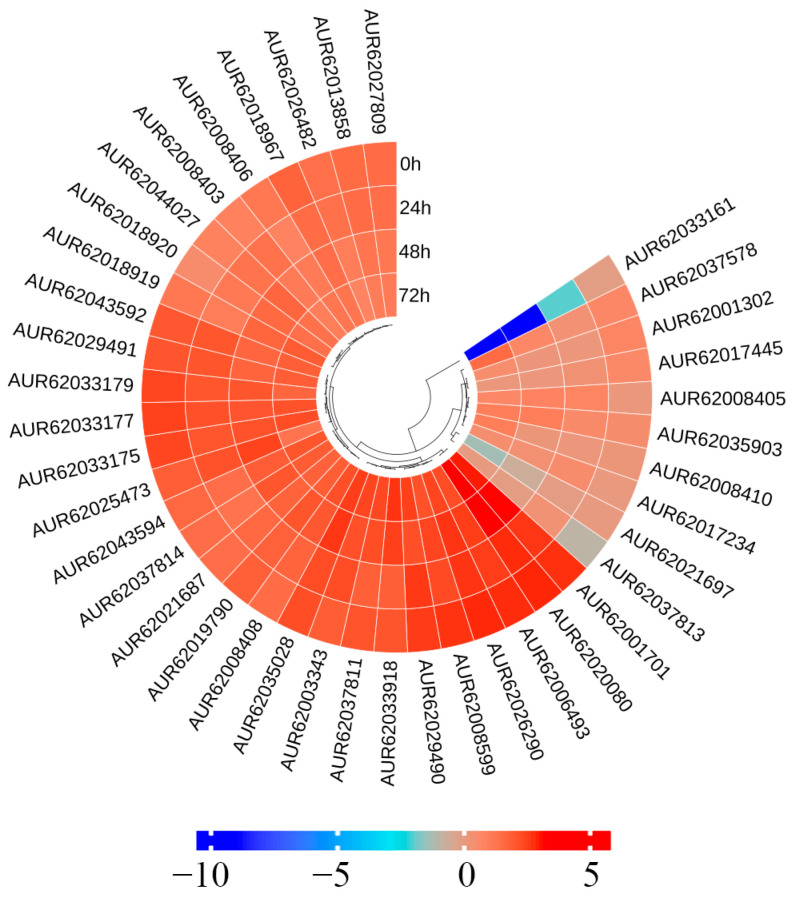
Heatmap of expression patterns of DEGs in glutathione metabolism pathway.

Photosynthesis decreased primarily between 24–48 h, and a simultaneously enriched pathway was “glutathione metabolism”, which is involved in the plant response to environmental stress, plant development and ROS signaling. Through pathway mapping localization, a total of 40 DEGs were found to participate in “glutathione metabolism” (Figure 10): five *APXs*, nine *GSTs*, thirteen *GSTUs*, two *Hsps*, two *CLEB3J9* and nine undefined (Table S2). Among them, *APXs*, *GSTs* and *GSTUs* all regulate ROS balance. Hsps can participate in the regulation of biological stresses as molecular chaperones, and the GO annotation for *CLEB3J9* is GO:0010206, which is involved in PS II process repair. High tolerance to salt stress in quinoa is speculated to be related to its efficient removal of excess ROS through “glutathione metabolism”.

### 3.10. Salt Stress Treatment Affects the Expression of Phenylpropanoid Biosynthesis, Starch and Sucrose Metabolism Genes in Quinoa Seedlings

**Figure 11:**
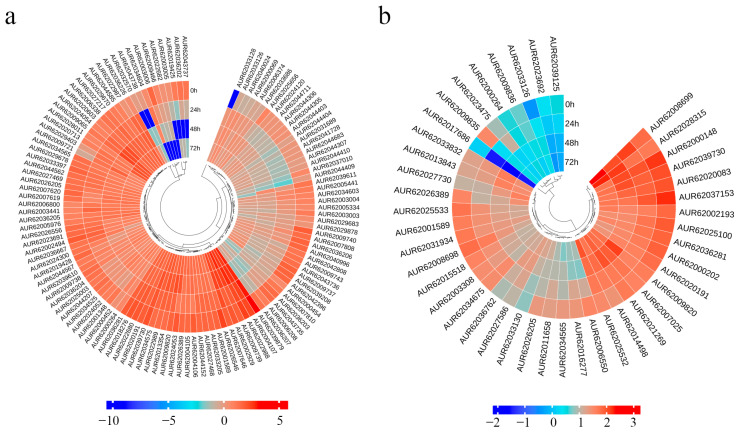
Heatmap of expression patterns of DEGs in phenylpropanoid biosynthesis (**a**) and starch and sucrose metabolism (**b**).

The KEGG mapper of DEGs identified early-term enrichment in the “phenylpropanoid biosynthesis” pathway and long-term enrichment in the “phenylpropanoid biosynthesis” and “starch and sucrose metabolism” pathways. Eighty-seven down-regulated and thirty-three up-regulated genes were mapped to the “phenylpropanoid biosynthesis” pathway (Figure 11a). These genes were annotated and found to contain primarily peroxidases (*PERs*), peroxisomal adenine nucleotide carrier 2 (*PNC2s*) and caffeic acid O-methyl transferase (*COMTs*) genes, which are generally involved in the elimination of H_2_O_2_, oxidation of toxic reducing agents, and biosynthesis and degradation of lignin (Table S3). There were 31 down-regulated and 16 up-regulated genes localized to “starch and sucrose metabolism” (Figure 11b). Four SS genes were identified by gene annotation in sixteen up-regulated genes (Table S4). Due to the less energy required for SS to catalyze sucrose catabolism, plants under stress conditions tend to rely more on sucrose synthase-catalyzed reactions to provide reducing sugars for normal development [33]. We speculate that quinoa seedlings resist salt stress primarily through “phenylpropanoid biosynthesis” and “starch and sucrose metabolism” processes in the late and long-term stages.

### 3.11. Transcription Factors in Quinoa Are Involved in Salt Stress Regulation

**Figure 12:**
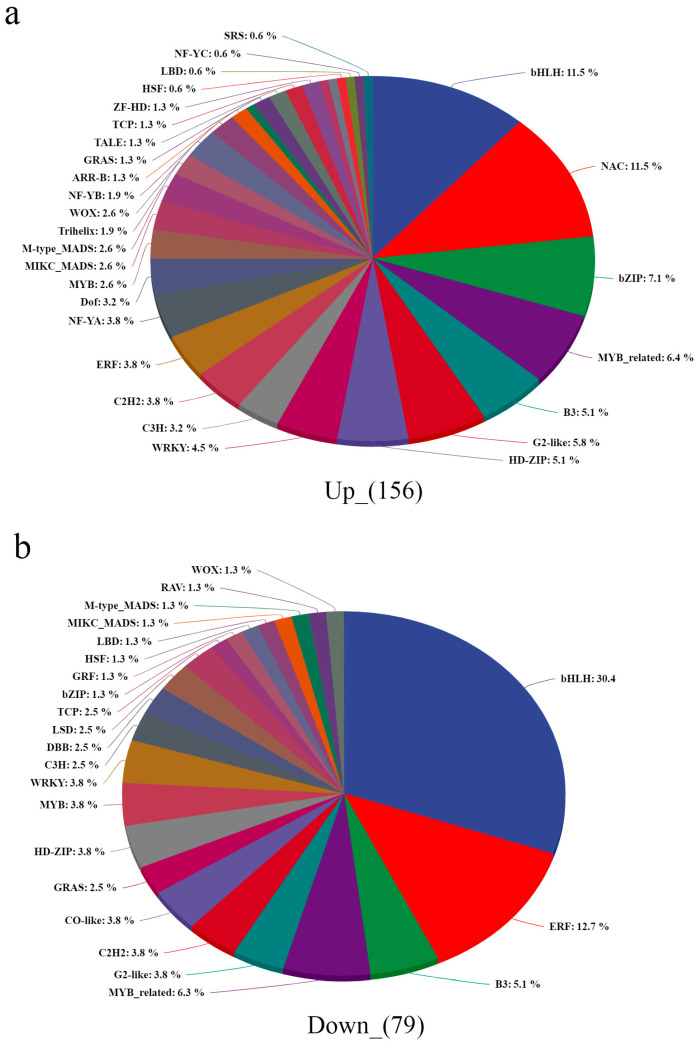
TFs in salt-induced (**a**) and repressed (**b**).

Due to the key role of transcription factors in the regulation of salt stress process sections, we predicted that TFs for 2411 and 2711 were up- and down-regulated DEGs, respectively. A total of 156 up-regulated TFs belonging to 31 TF families were screened and found to be dominated by *bHLH*, *NAC* and *bZIP*. Seventy-nine down-regulated TFs, belonging to twenty-three TF families, were dominated by *bHLH*, *ERF* and *B3* (Figure 12). *bHLH*, *ERF*, *B3*, *MYB*_related, *G2-like*, *C2H2*, *GRAS*, *HD-ZIP*, *MYB*, *WRKY*, *C3H*, *TCP*, *bZIP*, *GRF*, *HSF*, *LBD*, *MIKC_MADS*, *M-type_MADS* and *WOX* were shared between up- and down-regulated genes. Notably, we found that 45 TFs belonging to *NAC*, *NF-YA*, *Dof*, *Trihelix*, *NF-YB*, *ARR-B*, *GATA*, *TALE*, *ZF-HD*, *BES1*, *NF-YC* and *SRS* were specifically up-regulated by salt stress. Meanwhile, eight TFs belonging to *CO-like*, *DBB*, *LSD* and *RAV* were specifically down-regulated by salt stress (Tables S5 and S6). Therefore, we hypothesize that these TFs may be important positive and negative transcriptional regulators in response to salt stress signaling in quinoa seedlings.

### 3.12. Prediction and Overrepresented Cis-Elements in the Promoter Regions of Transcription Factors

**Figure 13:**
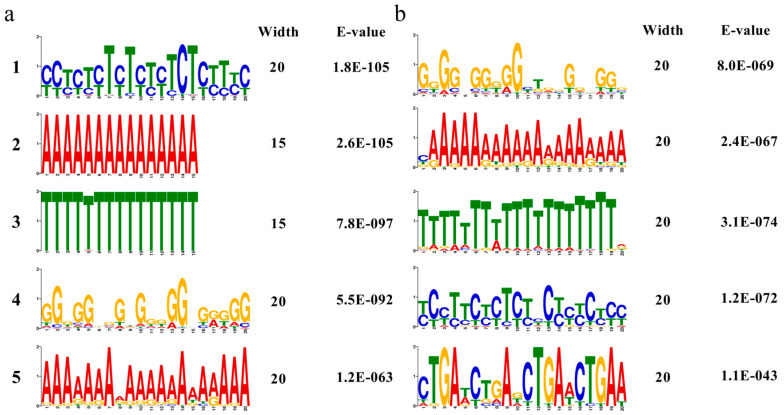
Cis−elements enriched in the promotors of salt-induced (**a**) and salt-repressed genes (**b**).

The transcriptional regulation of TFs underlying many biological processes is initiated by recognizing external signals, and deciphering the potential cis-elements of TFs affected by salt stress can help to rapidly identify upstream signals. To determine which cis-element is enriched in the TF promoters up- and down-regulated by salt stress, we submitted the 2.0 kb sequences of these gene promoter regions to PlantCARE and found that “defense and stress-responsive”, “MeJA-responsive”, “drought-inducibility”, “abscisic acid-responsive”, “auxin-responsive”, “salicylic acid-responsive”, “gibberellin-responsive” and other cis-elements were present in the 156 and 79 TF promoters that were up- and down-regulated (Figures S3 and S4). The 235 predicted TFs not only respond to abiotic stress regulation, but also participate in a variety of biogenic hormone metabolic pathways. Motif analysis of the promoter regions of TFs by MEME yielded five extremely significant DNA motifs for the up- and down-regulated TF promoter regions (Figure 13). With a cutoff of e-value = 0.1, we matched the overrepresented motifs to known cis-elements in Arabidopsis. Among the five motifs up-regulated by salt stress, motifs 1 and 5 were better matched with the *BPC5* binding site of the *BBRBPC* TFs, motifs 2 and 3 were better matched with the *VRN1* binding site of the *ABI3/VP1* TFs, and motif 4 was better matched with the *MYB67* binding site of the *MYB* TFs. VRNs are key regulators of the abscisic acid (ABA) signaling pathway, whereas *MYBs* are involved in the salicylic acid (SA) signaling pathway, both of which play an important role in plant responses to stresses. Among the five motifs down-regulated by salt stress, motifs 1 and 2 were better matched with the *MYB60* binding site of the *MYB* TFs, and motifs 3, 4 and 5 were better matched with the *DEL2* binding site of the *E2FDP* TFs, the *TCP1* binding site of the *TCP* TFs and the *WLIM2A* binding site of the *LIM* TFs (Table 5).

## 4. Discussion

Quinoa, an important model halophytic crop species, is used in grain production and is conducive to the development of saline agriculture, which not only promotes the utilization of arid and semiarid, saline-alkali and other wastelands, but also effectively supplements the production of staple food [34]. Despite a long history in studying the mechanisms of plant salinity tolerance, the engineering of salinity-tolerant crops remains very challenging. Crop salinity tolerance is a complex trait associated with multiple subtraits, each with a complex genetic basis [35]. Manipulating a single or a limited number of genes has proven to be inadequate to create salinity-tolerant crops. To alleviate this dilemma, engineering tolerant crops, which are typically minor crops cultivated in marginal areas, for better yields could be an easier alternative to engineering major crops to be salt tolerant, and quinoa is considered to be a perfect candidate [8]. Thus, it is essential to understand the mechanisms and explore the key regulatory factors of salt tolerance in quinoa.

Previously, numerous studies have demonstrated that higher NaCl stress can reduce the germination rate of quinoa and disrupt normal physiological processes [36,37]. In this study, the germination rate of quinoa seeds decreased with increasing NaCl concentration, and when NaCl exceeded 200 mM, the embryo and radicle length were significantly altered. In addition, the NaCl IC_50_ for germination rate was 312.8 mM, thus 300 mM NaCl was used for the simulated salt stress treatment of quinoa seedlings, and samples were collected at 0, 24, 48 and 72 h for MDA, H_2_O_2_, activities of antioxidant enzymes and RNA-seq analysis. In the experiment, we found that salt stress causes an imbalance of ROS in quinoa, which disrupts cell membrane integrity, whereas the elevated activity of antioxidant enzymes, such as SOD, POD, GR and GPX, can scavenge excess ROS and mitigate cell membrane damage. Transcriptome data analysis was performed, and clustering found that 0 and 24 h samples were clustered as adjacent clades, distant from the 48 and 72 h clades, consistent with the result of PCA between samples. We hypothesize that the mechanisms involved in the regulation of salt stress in quinoa may change as the duration of salt stress increases. Cross-group comparison revealed that the number of DEGs produced increased with increasing duration of salt stress. Clustering analysis of the expression of DEGs also revealed that 0 and 24 h were clustered into one clade and 48 and 72 h were clustered into another clade, providing further evidence of differences in the genes and biological processes involved in the regulation of quinoa in the early and late stages of salt stress. KEGG enrichment analysis indicated that a large number of key genes for photosynthesis were inhibited between 24~48 h, while the expression levels of key genes in glutathione metabolism were generally higher, suggesting that fine regulation of glutathione metabolic processes is a key pathway in quinoa for scavenging ROS in response to salt stress early stage [38]. In contrast, DEGs occurring in the late and long-term stages of salt stress were mainly enriched in “phenylpropanoid biosynthesis” and “starch and sucrose metabolism”. “Phenylpropanoid biosynthesis” is a series of enzymatic reactions starting with phenylalanine that produce a large number of secondary metabolites that play an important role in plant growth and development, as well as plant–environment interactions [39,40]. Gene annotation revealed primarily *PER*, *PNC2* and *COMT* genes, which are involved in the elimination of H_2_O_2_, oxidation of toxic reducing agents, and lignin biosynthesis and degradation. The lignin produced by the lignin pathway can not only provide mechanical support for plants, but also participate in the transport of water and minerals. In addition, the xylem is rapidly loaded with Na^+^ to regulate osmotic pressure, which is also an important mechanism by which quinoa resists salt stress [41]. These data reveal why quinoa has elevated antioxidant enzyme activity during exposure to salt stress. “Starch and sucrose metabolism” is also important for plants to cope with abiotic stresses, as the process of starch and sucrose metabolism not only scavenges excess ROS to maintain normal cellular redox reactions [11], but also produces soluble sugars that regulate osmotic pressure homeostasis [42]. Large amounts of sucrose are produced during starch and sucrose metabolism, and the enrichment of sucrose under salt stress is considered a long-term defense strategy for plants [43,44]. Differences in the expression pattern of DEGs produced by time-course analysis of salt stress may be due to a preadaptive response to salinity in quinoa, resulting in a constitutive overexpression of stress-related genes [45,46]. However, due to the many ecological types of quinoa, whether these traits are widespread in quinoa requires further research.

Transcription factor annotation results revealed that twelve families of TFs (*NAC*, *NF-YA*, *Dof*, *Trihelix*, *NF-YB*, *ARR-B*, *GATA*, *TALE*, *ZF-HD*, *BES1*, *NF-YC* and *SRS*) were specifically up-regulated and four families of TFs (*CO-like*, *DBB*, *LSD* and *RAV*) were specifically down-regulated in DEGs under salt stress, indicating that they play an important role in enhancing and reducing salt tolerance in quinoa. In addition, 20 families of TFs (*bHLH*, *ERF*, *B3*, *MYB_related*, *G2-like*, *C2H2*, *GRAS*, *HD-ZIP*, *MYB*, *WRKY*, *C3H*, *TCP*, *bZIP*, *GRF*, *HSF*, *LBD*, *MIKC_MADS*, *M-type*, *MADS* and *WOX*) were present in up- and down-regulated DEGs, suggesting possible antagonistic effects between different members of the same TF family in quinoa during resistance to salt stress. In recent years, many studies have also demonstrated that these transcription factors play an important role in the regulation of salt stress. An overexpression of *GmNAC06* can significantly increase the accumulation of proline and betaine in soybeans and Arabidopsis and can alleviate the oxidative stress caused by excess ROS, thereby enhancing plant salt tolerance [47]. *SmERF1* in *Eggplant* exhibits transcriptional activity in the nucleus, and knocking down the expression of *SmERF1* can significantly reduce antioxidant enzyme activity and improve salt sensitivity [48]. RAVs negatively regulate growth and multiple abiotic stresses in *Arabidopsis* [49]. In addition, bioinformatic analysis of the promoter regions of these TFs revealed an enrichment of diverse cis-acting regulatory elements associated with stress adaptation and phytohormone signaling. Notably, “defense and stress-responsive” and “drought-induvibility” elements were identified, suggesting roles in abiotic stress tolerance. Multiple hormone-related motifs were also detected, including “MeJA-responsive”, “abscisic acid-responsive”, “auxin-responsive”, “salicylic acid-responsive”, “gibberellin-responsive” and other action progenitors. The co-occurrence of these regulatory elements underscores the functional versatility of these TFs in salt stress and endogenous signals. The promoters of up-regulated TFs were significantly enriched for motifs, including *VRN1* (a vernalization-responsive element critical for flowering time control), *MYB67* (linked to abiotic stress responses and ion homeostasis) and *BPC5* (a GAGA-binding motif involved in chromatin remodeling and growth regulation). Conversely, promoters of down-regulated TFs were predominantly enriched for motifs such as *MYB60* (associated with drought response and stomatal closure), *TCP1* (a plant-specific motif regulating cell proliferation and leaf morphogenesis) and *DEL2* (implicated in secondary cell wall biosynthesis and developmental repression). This further suggests that quinoa may prioritize stress adaptation under salt stress conditions through its own growth and developmental transitions [50].

## 5. Conclusions

This study found that the growth of quinoa seedlings was significantly inhibited under salt stress, and physiological and biochemical traits such as H_2_O_2_, MDA, SOD, POD, GR and GPX showed an upward trend, suggesting that the elevated activity of antioxidant enzymes in seedlings can alleviate the damage caused by salt stress. In parallel, RNA-seq analysis showed that the ROS imbalance caused by early-term salt stress induced the up-regulation of the expression of antioxidant enzyme-related genes (e.g., *GSTs*, *GSTUs*, *APXs*, etc.) in the “glutathione metabolism” pathway. However, the expression of *SS* genes in the “starch and sucrose metabolism” pathway was up-regulated during the later stages of salt stress, suggesting that quinoa may also utilize sucrose synthase-catalyzed reactions that reduce energy consumption and provide reducing sugars for development. In addition, 235 TFs were predicted to be involved in the salt stress response, with multiple cis-elements in their promoter regions (e.g., “MeJA-responsive”, “abscisic acid-responsive”, “auxin-responsive”, etc.), which also suggests that there are multiple hormones involved in the salt stress response process in quinoa. Understanding these mechanisms not only enhances our knowledge of salt stress responses, but also offers potential strategies for improving quinoa salinity and productivity.

## Figures and Tables

**Table 1:** Summary of the RNA-seq data generated by Illumina sequencing in this study.

Sample	Total Bases	CG	Q30	Inputs Reads	Mapped Reads	Multiple Mapped	Uniquely Mapped
0 h-1	8,843,742,774	44.42%	94.04%	59,298,848	57,400,456 (96.80%)	3,921,182 (6.61%)	53,479,274 (90.19%)
0 h-2	10,255,249,580	44.35%	93.61%	67,999,860	65,990,183 (97.04%)	4,623,856 (6.80%)	61,366,327 (90.24%)
0 h-3	8,680,840,872	43.98%	93.74%	59,573,372	56,359,117 (94.60%)	3,395,622 (5.70%)	52,963,495 (88.90%)
24 h-1	7,918,433,584	44.53%	93.94%	52,369,708	50,951,859 (97.29%)	4,434,371 (8.47%)	46,517,488 (88.83%)
24 h-2	8,200,490,347	44.56%	93.09%	54,658,448	52,822,028 (96.64%)	4,634,431 (8.48%)	48,187,597 (88.16%)
24 h-3	8,226,565,958	44.88%	93.33%	52,652,994	50,838,650 (96.55%)	5,104,926 (9.70%)	45,733,724 (86.86%)
48 h-1	8,594,627,494	43.67%	93.84%	58,684,348	57,358,612 (97.74%)	3,938,468 (6.71%)	53,420,144 (91.03%)
48 h-2	8,227,452,486	44.32%	92.79%	56,137,574	54,107,863 (96.38%)	6,304,612 (11.23%)	47,803,251 (85.15%)
48 h-3	7,261,377,841	44.41%	92.95%	49,692,458	48,213,248 (97.02%)	4,596,286 (9.25%)	43,616,962 (87.77%)
72 h-1	7,089,095,616	44.31%	92.88%	48,094,656	46,794,677 (97.30%)	5,274,057 (10.97%)	41,520,620 (86.33%)
72 h-2	7,299,664,280	44.05%	92.27%	50,057,912	48,374,621 (96.64%)	6,299,653 (12.58%)	42,074,968 (84.05%)
72 h-3	6,901,494,782	44.80%	93.22%	47,039,728	45,724,732 (97.20%)	5,697,288 (12.11%)	40,027,444 (85.09%)

**Table 2:** The expressed genes with an average TPM > 1 in each group.

Group	Number of Expressed Genes (Average TPM > 1)
0 h	24,350
24 h	24,251
48 h	24,075
72 h	24,292

**Table 3:** The DEGs screened by comparison between groups.

	Up-Regulated	Down-Regulated
24 h vs. 0 h	435	643
48 h vs. 0 h	1350	1795
48 h vs. 24 h	524	588
72 h vs. 0 h	1553	1559
72 h vs. 24 h	348	503
72 h vs. 48 h	22	54

**Table 4:** KEGG enrichment analysis of the genes in different expression pattern clusters.

	KEGG Pathway	ID	*p* Value
Cluster 3	Phenylpropanoid biosynthesis	ko00940	4.58 × 10^−13^
	Zeatin biosynthesis	ko00908	2.75 × 10^−23^
	Glutathione metabolism	ko00480	0.002475429
Cluster 6	Phenylpropanoid biosynthesis	ko00940	0.002516425
Cluster 7	Photosynthesis-antenna proteins	ko00196	7.56 × 10^−7^
Cluster 9	Phenylpropanoid biosynthesis	ko00940	0.001351993
	Cysteine and methionine metabolism	ko00520	0.001256953
Cluster 12	Phenylpropanoid biosynthesis	ko00940	0.000255861
Cluster 15	Phenylpropanoid biosynthesis	ko00940	3.21 × 10^−8^
	Ubiquitin mediated proteolysis	ko04120	0.000542454
	RNA polymerase	ko03020	3.54 × 10^−12^
	Protein processing in endoplasmic reticulum	ko04141	1.25 × 10^−4^

**Table 5:** Overrepresented cis-elements in the promotors of TFs.

	Discovered Motif	Matched Known Motif in Arabidopsis	E-Value
Up-regulated	YYHNHYBYYYYYYYHYYYHH	BBRBPC_tnt.BPC5_colamp_a_m1 (BPC5)	1.54 × 10^−3^
	AAAAAAAAAAAAAAA	ABI3VP1_tnt.VRN1_col_a_m1 (VRN1)	2.71 × 10^−13^
	TTTTWTTTTTTTTTT	ABI3VP1_tnt.VRN1_col_a_m1 (VRN1)	5.17 × 10^−7^
	NVNNNDNDDNNDDRBDNDDV	MYB_tnt.MYB67_col_a_m1 (MYB67)	9.79 × 10^−3^
	VRRRDDDADDVNVWDHRRRA	BBRBPC_tnt.BPC5_colamp_a_m1 (BPC5)	1.24 × 10^−2^
	YYHNHYBYYYYYYYHYYYHH	BBRBPC_tnt.BPC5_colamp_a_m1 (BPC5)	1.54 × 10^−3^
Down-regulated	NNRNRNNNDKHNNNNDNDNN	MYB_tnt.MYB60_col_m1 (MYB60)	2.04 × 10^0^
	BRWRRRRDDDDDDDDRRNND	MYB_tnt.MYB60_col_m1 (MYB60)	3.07 × 10^−4^
	NDDWNDKWDWWHWWWWHTKD	E2FDP_tnt.DEL2_col_a_m1 (DEL2)	5.79 × 10^−1^
	HYHYNHHBHYHHYYBHNNNH	TCP_tnt.TCP1_col_a_m1 (TCP1)	3.74 × 10^−1^
	HKGADYBVADYTKADYYKAW	LIM_tnt.WLIM2A_col_a_m1 (WLIM2A)	9.65 × 10^0^

## Data Availability

The RNA-seq data generated from Chenododium quinoa seeding samples in this study were deposited in the NCBI SRA database (http://trace.ncbi.nlm.nih.gov/Traces/sra accessed on 6 January 2025) under accession PRJNA894199.

## Supplementary Materials

The following supporting information can be downloaded at https://www.mdpi.com/article/10.3390/biology14040416/s1: Table S1: The primer design of genes. Table S2: Annotation of DEGs in the glutathione metabolic pathway. Table S3: Annotation of DEGs in the phenylpropanoid biosynthesis pathway. Table S4: Annotation of DEGs in the starch and sucrose metabolism pathway. Tables S5 and S6: TFs in salt-repressed. Figure S1: Correlation analysis of QRT-PCR and TMP. Figure S2: Photographs of quinoa seedlings under CK and salt stress conditions. Figures S3 and S4: Cis-elements in the promoter regions of transcription factors.
