# Supplementary material for: Physiological Changes and Time-Course Transcriptomic Analysis of Salt Stress in Chenopodium quinoa
Source: Biology (Basel). 2025 Apr 13;14(4):416. doi: 10.3390/biology14040416 (PMC12024985; doi:10.3390/biology14040416)
Supplement: Supplementary file 1 [file biology-14-00416-s001.zip › Supplementary(Figure+Table)/Table S1.pdf]

Table S1 The Primer design of genes.

Gene ID	Forward primer for qRT-PCR（5'-3'）	Reverse primer for qRT-PCR（5'-3'）	Size of the fragment	Annealing temperature
AUR62033918	GGGTAGTCCCTTCAGTATGC	GCCTTTGTGCTTTGTTCTC	342	56.8
AUR62018919	CGGAGGAAACAACATTGG	GGCATTCATAAGGGCAAAG	226	57.0
AUR62001701	TGTTGGAGCAAGAGTTAGGG	GCTTTAGCCACACTTTCCC	193	57.5
AUR62026482	GGTTCCCAACATTTCGTG	CCTTGACAGCATCATCTGC	297	57.2
AUR62008408	AAAGGAAGGCAACCCATC	GAAAGGACTAAACCCAAAGAGC	205	58.5
AUR62044027	TGCTTGCGGTTGAAGTAAC	TTTGAAAGGGTCCTCTGTG	248	57.6
ACT-1	GTCCACAGAAAGTGCTTCTAAG	AACAACTCCTCACCTTCTCATG	194	58.0
