# Supplementary material for: Physiological Changes and Time-Course Transcriptomic Analysis of Salt Stress in Chenopodium quinoa
Source: Biology (Basel). 2025 Apr 13;14(4):416. doi: 10.3390/biology14040416 (PMC12024985; doi:10.3390/biology14040416)
Supplement: Supplementary file 1 [file biology-14-00416-s001.zip › Supplementary(Figure+Table)/Table S2.pdf]

Table S2 Annotation of DEGs in the glutathione metabolic pathway.

Symbol	Gene ID
APXs	*AUR62008599*，*AUR62008408*，*AUR62043594*，*AUR62044027*，*AUR62037814*
CLEB3J9	*AUR62033175*，*AUR62008405*
GSTs	*AUR62033918*，*AUR62018967*，*AUR62018920*，*AUR62006493*，*AUR62026482*，*AUR62033179*，*AUR62037811*，*AUR62018919*
GSTUs	*AUR62001302*，*AUR62027809*，*AUR62025473*，*AUR62008403*，*AUR62029490*，*AUR62035028*，*AUR62021697*，*AUR62029491*，*AUR62033161*，*AUR62001701*，*AUR62037813*，*AUR62013858*，*AUR62043592*，*AUR62021687*
Hsp	*AUR62020080*，*AUR62037578*
