# Supplementary material for: Physiological Changes and Time-Course Transcriptomic Analysis of Salt Stress in Chenopodium quinoa
Source: Biology (Basel). 2025 Apr 13;14(4):416. doi: 10.3390/biology14040416 (PMC12024985; doi:10.3390/biology14040416)
Supplement: Supplementary file 1 [file biology-14-00416-s001.zip › Supplementary(Figure+Table)/Table S3.pdf]

Table S3 Annotation of DEGs in the phenylpropanoid biosynthesis pathway.

Gene ID	Symbol	Gene ID	Symbol	Gene ID	Symbol
AUR62000069	BGLU2	*AUR62001589*	BoGH3B	*AUR62003005*	PER60
AUR62000264	BGLU42	*AUR62002494*	CYP84A1	*AUR62003006*	PNC1
AUR62000452	PER5	*AUR62002929*	PER57	*AUR62003441*	PER55
AUR62000454	PER24	*AUR62003003*	PER57	*AUR62004105*	PER20
AUR62001348	PER60	*AUR62003004*	PER2	*AUR62004106*	PER4
AUR62004107	PER72	*AUR62006800*	PER5	*AUR62009466*	SHT
AUR62005334	CYP84A1	*AUR62007619*	PER20	*AUR62009721*	PER2
AUR62005441	CYP98A2	*AUR62007620*	PER4	*AUR62009737*	PNC2
AUR62005976	PER15	*AUR62007646*	CYP84A1	*AUR62009738*	-
AUR62006208	PER60	*AUR62007808*	PER57	*AUR62009739*	PER2
AUR62006338	PNC1	*AUR62007810*	PER44	*AUR62009740*	PER60
AUR62006374	PER53	*AUR62009065*	PER45	*AUR62009743*	PER57
AUR62009820	BGLU44	*AUR62022987*	-	*AUR62025656*	PER60
AUR62013354	PER25	*AUR62022988*	PER4	*AUR62026205*	BGLU24
AUR62018276	CAD6	*AUR62022989*	PER15	*AUR62026389*	BGLU24
AUR62019425	PER71	*AUR62023691*	CYP84A1	*AUR62026546*	PER57
AUR62019428	PNC2	*AUR62024052*	PER	*AUR62026556*	HCBT3
AUR62020191	BoGH3B	*AUR62024053*	PER	*AUR62027217*	PER17
AUR62020603	PER45	*AUR62024054*	PER4	*AUR62027468*	COMTs
AUR62020713	CYP84A1	*AUR62024120*	-	*AUR62027469*	COMTs
AUR62022680	COMT1	*AUR62024300*	CYP98A3	*AUR62028011*	CYP84A1
AUR62022682	-	*AUR62025003*	PER27	*AUR62029403*	PER55
AUR62029670	PNC1	*AUR62033126*	-	*AUR62034565*	BGLU11
AUR62029678	PER65	*AUR62033128*	-	*AUR62034575*	COMT1
AUR62029683	-	*AUR62033205*	4CL1	*AUR62034603*	PER39
AUR62029878	COMTs	*AUR62033397*	ALDH2C4	*AUR62034604*	PER24
AUR62031689	CYP98A3	*AUR62033686*	COMT1	*AUR62036202*	PER60
AUR62032570	CYP98A2	*AUR62034525*	PER9	*AUR62036203*	PER54
AUR62039730	BoGH3B	*AUR62044207*	PER4	*AUR62044711*	PER4
AUR62039610	PER39	*AUR62043738*	-	*AUR62044585*	PNC1
AUR62039611	PER27	*AUR62044152*	4CL1	*AUR62044683*	PER4
AUR62036281	BoGH3B	*AUR62042808*	PER7	AUR62044409	-
AUR62036204	PER3	*AUR62039879*	PAL	*AUR62044305*	PER4

AUR62036205	-	*AUR62040024*	PER21	*AUR62044306*	PER
AUR62036206	-	*AUR62040996*	PER7	*AUR62044307*	-
AUR62036207	PNC2	*AUR62041728*	PER4	*AUR62044403*	PER4
AUR62036226	PNC1	*AUR62042396*	PER56	*AUR62044404*	APXT
AUR62036667	PER24	*AUR62043735*	PER60	*AUR62044410*	PER4
AUR62037010	PER60	*AUR62043736*	PER57	*AUR62044562*	4CL1
AUR62039208	PNC2	*AUR62043737*	-	*AUR62044567*	4CL1
