# Supplementary material for: Physiological Changes and Time-Course Transcriptomic Analysis of Salt Stress in Chenopodium quinoa
Source: Biology (Basel). 2025 Apr 13;14(4):416. doi: 10.3390/biology14040416 (PMC12024985; doi:10.3390/biology14040416)
Supplement: Supplementary file 1 [file biology-14-00416-s001.zip › Supplementary(Figure+Table)/Table S4.pdf]

Table S4 Annotation of DEGs in the starch and sucrose metabolism pathway.

Gene ID	Symbol	Gene ID	Symbol	Gene ID	Symbol
AUR62017685	-	*AUR62013843*	AMY2	*AUR62009836*	CWINV1
AUR62017686	-	*AUR62023692*	APS2	*AUR62009835*	CWINV2
AUR62008698	-	*AUR62000148*	BAM1	*AUR62039125*	CWINV3
AUR62025533	-	*AUR62011658*	BAM2	*AUR62016277*	DPEP
AUR62025100	-	*AUR62034565*	BGLU11	*AUR62003308*	DPEP
AUR62002193	-	*AUR62000069*	BGLU2	*AUR62031934*	HXK1
AUR62033126	-	*AUR62026389*	BGLU24	*AUR62033832*	HXK2
AUR62033128	-	*AUR62026205*	BGLU24	*AUR62033130*	SK36
AUR62021269	-	*AUR62000264*	BGLU42	*AUR62028315*	SS
AUR62007025	-	*AUR62009820*	BGLU44	*AUR62008699*	SS1
AUR62034675	ADG2	*AUR62001589*	BoGH3B	*AUR62025532*	SS1
AUR62037153	AGPB1	*AUR62036281*	BoGH3B	*AUR62027586*	SS4
AUR62014498	AGPS1	*AUR62039730*	BoGH3B	*AUR62015518*	TIV1
AUR62020083	α-GPs	*AUR62020191*	BoGH3B	*AUR62027730*	TPPD
AUR62012979	AMY1.3	*AUR62006550*	CEL1	*AUR62023475*	TPPH
AUR62036762	AMY2	*AUR62000202*	CEL1		
