# Supplementary material for: Physiological Changes and Time-Course Transcriptomic Analysis of Salt Stress in Chenopodium quinoa
Source: Biology (Basel). 2025 Apr 13;14(4):416. doi: 10.3390/biology14040416 (PMC12024985; doi:10.3390/biology14040416)
Supplement: Supplementary file 1 [file biology-14-00416-s001.zip › Supplementary(Figure+Table)/Table S5.pdf]

Table S5 TFs in salt-respressed.

| Gene ID | TFs family | Best hit in A.thaliana | E-value | Description |
|---|---|---|---|---|
| *AUR62043211* | bHLH | AT3G50330.1 | 5e-49 | bHLH family protein |
| *AUR62003315* | B3 | AT4G33280.1 | 5e-19 | B3 family protein |
| *AUR62016620* | B3 | AT3G19184.1 | 1e-67 | B3 family protein |
| *AUR62018428* | B3 | AT4G01500.1 | 1e-06 | B3 family protein |
| *AUR62022423* | B3 | AT5G42700.1 | 5e-66 | B3 family protein |
| *AUR62001913* | bHLH | AT4G36930.1 | 4e-54 | bHLH family protein |
| *AUR62003896* | bHLH | AT5G67110.3 | 9e-22 | bHLH family protein |
| *AUR62004246* | bHLH | AT4G37850.1 | 2e-39 | bHLH family protein |
| *AUR62004248* | bHLH | AT4G37850.1 | 3e-38 | bHLH family protein |
| *AUR62004545* | bHLH | AT4G00870.1 | 3e-70 | bHLH family protein |
| *AUR62005874* | bHLH | AT2G22760.1 | 4e-38 | bHLH family protein |
| *AUR62005875* | bHLH | AT4G37850.1 | 2e-48 | bHLH family protein |
| *AUR62005877* | bHLH | AT4G37850.1 | 4e-44 | bHLH family protein |
| *AUR62011907* | bHLH | AT4G16430.1 | 1e-146 | bHLH family protein |
| *AUR62014013* | bHLH | AT4G37850.1 | 1e-49 | bHLH family protein |
| *AUR62021282* | bHLH | AT5G51790.1 | 6e-22 | bHLH family protein |
| *AUR62023232* | bHLH | AT5G37800.1 | 1e-55 | Rhd Six-Like 1 |
| *AUR62031273* | bHLH | AT5G67060.1 | 2e-49 | bHLH family protein |
| *AUR62031976* | bHLH | AT3G21330.1 | 3e-58 | bHLH family protein |
| *AUR62032870* | bHLH | AT4G37850.1 | 2e-48 | bHLH family protein |
| *AUR62038707* | bHLH | AT4G33880.1 | 6e-59 | Root Hair Defective 6 |
| *AUR62038708* | bHLH | AT2G14760.1 | 2e-31 | bHLH family protein |
| *AUR62038798* | bHLH | AT4G33880.1 | 7e-51 | Root Hair Defective 6 |
| *AUR62038801* | bHLH | AT4G33880.1 | 2e-52 | Root Hair Defective 6 |
| *AUR62042990* | bHLH | AT2G14760.1 | 1e-58 | bHLH family protein |
| *AUR62030640* | bZIP | AT5G11260.1 | 4e-66 | bZIP family protein |
| *AUR62007281* | C2H2 | AT4G17810.1 | 5e-25 | C2H2 family protein |
| *AUR62018723* | C2H2 | AT4G17810.1 | 2e-30 | C2H2 family protein |
| *AUR62038383* | C2H2 | AT2G28710.1 | 5e-35 | C2H2 family protein |
| *AUR62009210* | C3H | AT4G29190.1 | 1e-100 | C3H family protein |
| *AUR62040207* | C3H | AT2G19810.1 | 1e-102 | C3H family protein |
| *AUR62009440* | CO-like | AT3G02380.1 | 1e-140 | CONSTANS-like 2 |
| *AUR62030805* | CO-like | AT1G25440.1 | 4e-85 | zinc finger protein |

| | | | | |
|---|---|---|---|---|
| *AUR62035221* | CO-like | AT1G25440.1 | 1e-58 | zinc finger protein |
| *AUR62014102* | DBB | AT5G48250.1 | 8e-31 | zinc finger protein |
| *AUR62014436* | DBB | AT1G75540.1 | 2e-74 | Salt tolerance homolog2 |
| *AUR62002115* | ERF | AT3G23240.1 | 2e-52 | ERF family protein |
| *AUR62014912* | ERF | AT4G18450.1 | 2e-41 | ERF family protein |
| *AUR62015277* | ERF | AT5G19790.1 | 2e-38 | Related to AP2 |
| *AUR62018592* | ERF | AT4G13620.1 | 5e-54 | ERF family protein |
| *AUR62019829* | ERF | AT1G64380.1 | 2e-63 | ERF family protein |
| *AUR62020826* | ERF | AT4G13620.1 | 1e-61 | ERF family protein |
| *AUR62024721* | ERF | AT3G23240.1 | 2e-55 | Ethylene response factor |
| *AUR62025997* | ERF | AT3G57600.1 | 2e-79 | ERF family protein |
| *AUR62039694* | ERF | AT5G11590.1 | 7e-58 | ERF family protein |
| *AUR62040909* | ERF | AT5G19790.1 | 5e-49 | Related to AP2 |
| *AUR62021010* | G2-like | AT5G06800.2 | 5e-05 | G2-like family protein |
| *AUR62027330* | G2-like | AT5G45580.1 | 5e-41 | G2-like family protein |
| *AUR62040096* | G2-like | AT5G42630.1 | 2e-21 | G2-like family protein |
| *AUR62001765* | GRAS | AT2G29060.1 | 1e-161 | GRAS family protein |
| *AUR62040261* | GRAS | AT3G49950.1 | 1e-151 | GRAS family protein |
| *AUR62001481* | GRF | AT3G13960.1 | 2e-49 | Growth-regulating factor |
| *AUR62026453* | HD-ZIP | AT3G01470.1 | 3e-34 | Homeobox 1 |
| *AUR62022751* | HD-ZIP | AT4G00730.1 | 0.0 | HD-ZIP family protein |
| *AUR62021220* | HD-ZIP | AT4G00730.1 | 0.0 | HD-ZIP family protein |
| *AUR62029043* | HSF | AT2G26150.1 | 6e-21 | Heat shock factor |
| *AUR62022945* | LBD | AT2G30130.1 | 1e-77 | LBD family protein |
| *AUR62018732* | LSD | AT1G32540.3 | 6e-21 | LSD one like 1 |
| *AUR62023662* | M-type_MADS | AT5G48670.1 | 6e-34 | AGAMOUS-like 80 |
| *AUR62026426* | MIKC_MADS | AT3G57230.1 | 4e-79 | AGAMOUS-like 16 |
| *AUR62017737* | MYB | AT5G08520.1 | 6e-65 | MYB family protein |
| *AUR62021199* | MYB | AT4G25560.1 | 5e-60 | MYB domain protein |
| *AUR62041339* | MYB | AT2G37630.1 | 1e-160 | MYB family protein |
| *AUR62001769* | MYB_related | AT1G07540.1 | 1e-130 | TRF-like 2 |
| *AUR62016741* | MYB_related | AT1G75250.1 | 1e-37 | RAD-like 6 |
| *AUR62020926* | MYB_related | AT1G08810.1 | 9e-29 | MYB domain protein 60 |
| *AUR62044763* | MYB_related | AT2G37630.1 | 1e-136 | MYB family protein |
| *AUR62035279* | RAV | AT1G13260.1 | 1e-141 | Related to ABI3/VP1 1 |
| *AUR62019560* | TCP | AT1G53230.1 | 1e-86 | PCF transcription factor 3 |
| *AUR62017610* | WOX | AT3G18010.1 | 2e-40 | Related homeobox 1 |
| *AUR62004466* | WRKY | AT4G11070.1 | 7e-44 | WRKY family protein |
| *AUR62019820* | WRKY | AT4G11070.1 | 3e-55 | WRKY family protein |
| *AUR62024642* | WRKY | AT1G29280.1 | 5e-35 | WRKY family protein |
| *AUR62017336* | bHLH | AT4G25410.1 | 2e-28 | bHLH family protein |
| *AUR62027424* | bHLH | AT5G67060.1 | 5e-50 | bHLH family protein |

| | | | | |
|---|---|---|---|---|
| *AUR62032868* | bHLH | AT4G37850.1 | 1e-36 | bHLH family protein |
| *AUR62007267* | LSD | AT1G32540.3 | 2e-81 | LSD one like 1 |
| *AUR62018493* | MYB_related | AT1G08810.1 | 6e-28 | MYB domain protein 60 |
| *AUR62005756* | TCP | AT1G69690.1 | 9e-48 | TCP family protein |
