# Supplementary material for: Physiological Changes and Time-Course Transcriptomic Analysis of Salt Stress in Chenopodium quinoa
Source: Biology (Basel). 2025 Apr 13;14(4):416. doi: 10.3390/biology14040416 (PMC12024985; doi:10.3390/biology14040416)
Supplement: Supplementary file 1 [file biology-14-00416-s001.zip › Supplementary(Figure+Table)/Table S6.pdf]

Table S6 TFs in salt-induced.

Gene ID	TFs family	Best hit in *A.thaliana*	E-value	Description
AUR62009509	bHLH	AT5G50915.2	2e-58	bHLH family protein
AUR62001964	G2-like	AT4G37180.1	3e-44	G2-like family protein
AUR62020634	NAC	AT2G24430.2	1e-107	NAC
AUR62038748	NAC	AT2G24430.2	1e-106	NAC
AUR62024182	ARR-B	AT1G67710.1	1e-124	Response regulator 11
AUR62033367	ARR-B	AT4G31920.1	9e-14	response regulator 10
AUR62003318	B3	AT4G33280.1	2e-18	B3 family protein
AUR62006873	B3	AT4G33280.1	1e-16	B3 family protein
AUR62009734	B3	AT2G30470.1	0.0	Sugar-inducible gene 2
AUR62020518	B3	AT4G33280.1	2e-05	B3 family protein
AUR62031619	B3	AT5G18000.1	1e-06	Verdandi
AUR62036210	B3	AT2G30470.1	0.0	Sugar-inducible gene 2
AUR62037841	B3	AT3G26790.1	5e-86	B3 family protein
AUR62039039	B3	AT4G33280.1	8e-17	B3 family protein
AUR62039493	BES1	AT1G78700.1	1e-110	BES1/BZR1 homolog 4
AUR62001169	bHLH	AT3G07340.1	2e-98	bHLH family protein
AUR62013093	bHLH	AT3G19860.1	3e-77	bHLH family protein
AUR62017206	bHLH	AT4G37850.1	9e-43	bHLH family protein
AUR62022910	bHLH	AT2G40200.1	1e-46	bHLH family protein
AUR62024616	bHLH	AT1G71200.1	6e-28	bHLH family protein
AUR62025963	bHLH	AT2G42280.1	3e-56	bHLH family protein
AUR62029874	bHLH	AT1G73830.1	4e-57	BR enhanced expression 3
AUR62030326	bHLH	AT3G56970.1	9e-50	bHLH family protein
AUR62037320	bHLH	AT1G25330.1	5e-56	bHLH family protein
AUR62039664	bHLH	AT5G08130.6	2e-90	bHLH family protein
AUR62043582	bHLH	AT2G42280.1	1e-59	bHLH family protein
AUR62044545	bHLH	AT5G50915.2	1e-57	bHLH family protein
AUR62005989	bZIP	AT1G75390.1	3e-44	Basic leucine-zipper 44
AUR62008004	bZIP	AT4G36730.1	1e-107	G-box binding factor 1
AUR62009445	bZIP	AT3G30530.1	4e-56	Basic leucine-zipper 42
AUR62016851	bZIP	AT4G38900.3	1e-160	bZIP family protein
AUR62020638	bZIP	AT5G24800.1	5e-50	Basic leucine zipper 9
AUR62030316	bZIP	AT5G06950.4	1e-162	bZIP family protein
AUR62031478	bZIP	AT2G46270.1	6e-71	G-box binding factor 3
AUR62033525	bZIP	AT1G19490.1	5e-32	bZIP family protein
AUR62038742	bZIP	AT5G24800.1	1e-45	Basic leucine zipper 9
AUR62043508	bZIP	AT1G77920.1	1e-131	bZIP family protein
AUR62003535	C2H2	AT1G30970.1	1e-121	C2H2 family protein
AUR62009773	C2H2	AT1G08290.1	3e-87	WIP domain protein 3
AUR62011972	C2H2	AT4G02670.1	1e-111	ID-domain 12
AUR62039122	C2H2	AT1G55110.1	1e-112	ID-domain 7
AUR62039219	C2H2	AT1G08290.1	4e-87	WIP domain protein

AUR62000818	C3H	AT5G44260.1	7e-57	C3H family protein
AUR62006795	C3H	AT5G26749.1	7e-41	C3H family protein
AUR62009376	C3H	AT3G08505.1	1e-141	C3H family protein
AUR62026005	C3H	AT2G41900.1	0.0	C3H family protein
AUR62043445	C3H	AT2G47850.1	1e-142	C3H family protein
AUR62014301	Dof	AT2G34140.1	5e-23	Dof family protein
AUR62016967	Dof	AT1G28310.2	4e-30	Dof family protein
AUR62022735	Dof	AT4G24060.1	8e-49	Dof family protein
AUR62027677	Dof	AT1G29160.1	7e-45	Dof family protein
AUR62005714	ERF	AT5G13330.1	4e-50	Related to AP2
AUR62011530	ERF	AT1G15360.1	8e-64	ERF family protein
AUR62017052	ERF	AT5G44210.1	8e-30	ERF domain protein
AUR62034546	ERF	AT1G75490.1	1e-51	ERF family protein
AUR62003842	G2-like	AT1G49560.1	1e-32	G2-like family
AUR62025961	G2-like	AT3G04030.3	1e-113	G2-like family
AUR62026360	G2-like	AT1G79430.2	8e-97	G2-like family
AUR62032797	G2-like	AT3G04030.3	2e-54	G2-like family
AUR62033426	G2-like	AT2G03500.1	1e-108	G2-like family
AUR62041489	G2-like	AT1G79430.2	3e-55	G2-like family
AUR62000171	GRAS	AT1G50420.1	0.00	Scarecrow-like 3
AUR62007068	GRF	AT4G24150.1	4e-37	Growth-regulating
AUR62027204	HD-ZIP	AT4G40060.1	2e-80	Homeobox protein
AUR62031613	HD-ZIP	AT5G15150.1	4e-61	Homeobox 3
AUR62026261	HD-ZIP	AT3G01470.1	1e-35	Homeobox 1
AUR62001044	HD-ZIP	AT5G65310.2	6e-41	Homeobox protein
AUR62035595	HD-ZIP	AT2G46680.2	7e-49	Homeobox 7
AUR62007058	HD-ZIP	AT3G61890.1	3e-40	Homeobox 12
AUR62019921	HD-ZIP	AT3G61890.1	3e-38	Homeobox 12
AUR62006027	HD-ZIP	AT2G46680.2	2e-36	Homeobox 7
AUR62035755	HSF	AT1G46264.1	1e-111	Heat shock protein
AUR62030617	LBD	AT1G31320.1	7e-68	LOB domain
AUR62019123	MADS	AT3G57230.2	5e-33	AGAMOUS-like
AUR62034318	MADS	AT1G71692.1	1e-36	AGAMOUS-like
AUR62039675	MADS	AT5G60910.1	2e-35	AGAMOUS-like
AUR62044546	MADS	AT5G48670.1	2e-49	AGAMOUS-like
AUR62009424	MADS	AT5G13790.1	5e-74	AGAMOUS-like
AUR62014442	MADS	AT3G54340.1	3e-74	MIKC_MADS family
AUR62027090	MADS	AT3G57230.1	2e-54	AGAMOUS-like
AUR62034559	MADS	AT3G54340.1	3e-72	MIKC_MADS family
AUR62017171	MYB	AT5G56110.1	1e-116	MYB domain protein
AUR62025185	MYB	AT3G13540.1	2e-62	MYB domain protein
AUR62039812	MYB	AT3G28910.1	1e-109	MYB domain protein
AUR62001838	MYB_related	AT5G59780.3	2e-58	MYB domain protein
AUR62004127	MYB_related	AT3G60460.1	7e-27	MYB family protein

AUR62004570	MYB_related	AT1G01060.3	2e-70	MYB_related family
AUR62009626	MYB_related	AT5G59780.3	1e-57	MYB domain protein
AUR62018777	MYB_related	AT5G45420.1	5e-64	MYB_related family
AUR62019621	MYB_related	AT1G17950.1	4e-19	MYB domain protein
AUR62023607	MYB_related	AT1G09770.1	0.0	Cell division cycle 5
AUR62031911	MYB_related	AT3G16350.1	6e-61	MYB_related family
AUR62003400	NAC	AT1G65910.1	1e-136	NAC domain protein
AUR62005686	NAC	AT5G13180.1	1e-67	NAC domain protein
AUR62005821	NAC	AT1G69490.1	2e-91	NAC, activated by AP3/PI
AUR62006223	NAC	AT5G22380.1	8e-66	NAC domain protein
AUR62007247	NAC	AT4G17980.1	5e-98	NAC domain protein
AUR62008438	NAC	AT5G61430.1	1e-139	NAC domain protein
AUR62013541	NAC	AT1G79580.1	1e-105	NAC family protein
AUR62016954	NAC	AT1G61110.1	7e-92	NAC domain protein
AUR62018752	NAC	AT4G17980.1	2e-98	NAC domain protein
AUR62029330	NAC	AT3G15510.1	5e-98	NAC domain protein
AUR62033276	NAC	AT4G29230.1	1e-143	NAC domain protein
AUR62001502	NF-YA	AT1G72830.1	9e-58	Nuclear factor, YA3
AUR62009388	NF-YA	AT5G12840.2	9e-47	Nuclear factor, YA1
AUR62009868	NF-YA	AT1G72830.1	3e-59	Nuclear factor, YA3
AUR62025174	NF-YA	AT1G54160.1	7e-45	Nuclear factor, YA5
AUR62029002	NF-YA	AT1G72830.1	8e-47	Nuclear factor, YA3
AUR62034487	NF-YA	AT5G06510.2	1e-20	Nuclear factor, YA10
AUR62021825	NF-YB	AT5G47670.2	9e-69	Nuclear factor, YB6
AUR62043264	NF-YB	AT2G47810.1	9e-62	Nuclear factor, YB5
AUR62000893	NF-YC	AT1G08970.3	8e-86	Nuclear factor, YC9
AUR62007636	SRS	AT3G51060.1	5e-45	(LRP) protein-related
AUR62003417	TCP	AT3G27010.1	7e-47	TCP family protein
AUR62040457	TCP	AT1G69690.1	5e-37	TCP family protein
AUR62001029	Trihelix	AT5G28300.1	1e-71	Trihelix family protein
AUR62032637	Trihelix	AT3G54390.1	1e-99	Trihelix family protein
AUR62044655	Trihelix	AT5G28300.1	2e-37	Trihelix family protein
AUR62031363	WOX	AT1G46480.1	2e-71	WOX family protein
AUR62002048	WOX	AT4G35550.1	2e-61	WOX family protein
AUR62003373	WOX	AT2G17950.1	2e-21	WOX family protein
AUR62001049	WRKY	AT5G28650.1	1e-103	WRKY family protein
AUR62003119	WRKY	AT1G69310.2	2e-68	WRKY family protein
AUR62006427	WRKY	AT3G56400.1	3e-27	WRKY family protein
AUR62007938	WRKY	AT1G69310.2	3e-72	WRKY family protein
AUR62030260	WRKY	AT4G18170.1	7e-60	WRKY family protein
AUR62008414	ZF-HD	AT1G74660.1	2e-39	Mini zinc finger 1
AUR62004153	bHLH	AT4G00050.1	1e-54	bHLH family protein
AUR62005699	bHLH	AT1G68920.3	7e-33	bHLH family protein
AUR62007060	bHLH	AT4G00870.1	9e-31	bHLH family protein

AUR62016510	bHLH	AT4G29100.1	1e-37	bHLH family protein
AUR62018341	bHLH	AT3G56970.1	2e-46	bHLH family protein
AUR62024650	bZIP	AT2G46270.1	2e-08	G-box binding factor 3
AUR62001996	C2H2	AT1G34370.3	2e-41	C2H2 family protein
AUR62004520	Dof	AT4G24060.1	4e-49	Dof family protein
AUR62002113	ERF	AT3G23230.1	2e-46	ERF family protein
AUR62016352	ERF	AT1G15360.1	3e-61	ERF family protein
AUR62005806	G2-like	AT3G04030.2	2e-55	G2-like family protein
AUR62043017	G2-like	AT1G79430.2	1e-59	G2-like family protein
AUR62016814	GATA	AT5G66320.2	7e-53	GATA transcription factor
AUR62033433	GRAS	AT1G14920.1	1e-130	GRAS family protein
AUR62042086	MYB	AT5G04760.1	6e-71	MYB family protein
AUR62016928	MYB_related	AT2G42150.1	9e-34	Bomodomain-protein
AUR62035366	MYB_related	AT3G24310.1	4e-50	MYB domain protein
AUR62000898	NAC	AT3G04070.2	2e-24	NAC domain protein
AUR62006836	NAC	AT5G64060.1	3e-62	NAC domain protein
AUR62014768	NAC	AT1G61110.1	1e-106	NAC domain protein
AUR62018102	NAC	AT5G61430.1	1e-107	NAC domain protein
AUR62029331	NAC	AT3G15510.1	1e-102	NAC domain protein
AUR62033188	NF-YB	AT2G47810.1	1e-62	Nuclear factor, YB5
AUR62020229	TALE	AT2G35940.1	1e-126	BEL1-like homeodomain
AUR62029222	TALE	AT2G27990.1	9e-88	BEL1-like homeodomain
AUR62003747	WOX	AT4G35550.1	2e-88	WUSCHEL related
AUR62005858	WRKY	AT2G03340.1	1e-152	WRKY family protein
AUR62011795	WRKY	AT1G29280.1	1e-38	WRKY family protein
AUR62021682	ZF-HD	AT1G74660.1	2e-39	Mini zinc finger
