# Supplementary figures and images for: Physiological Changes and Time-Course Transcriptomic Analysis of Salt Stress in Chenopodium quinoa
Source: Biology (Basel). 2025 Apr 13;14(4):416. doi: 10.3390/biology14040416 (PMC12024985; doi:10.3390/biology14040416)

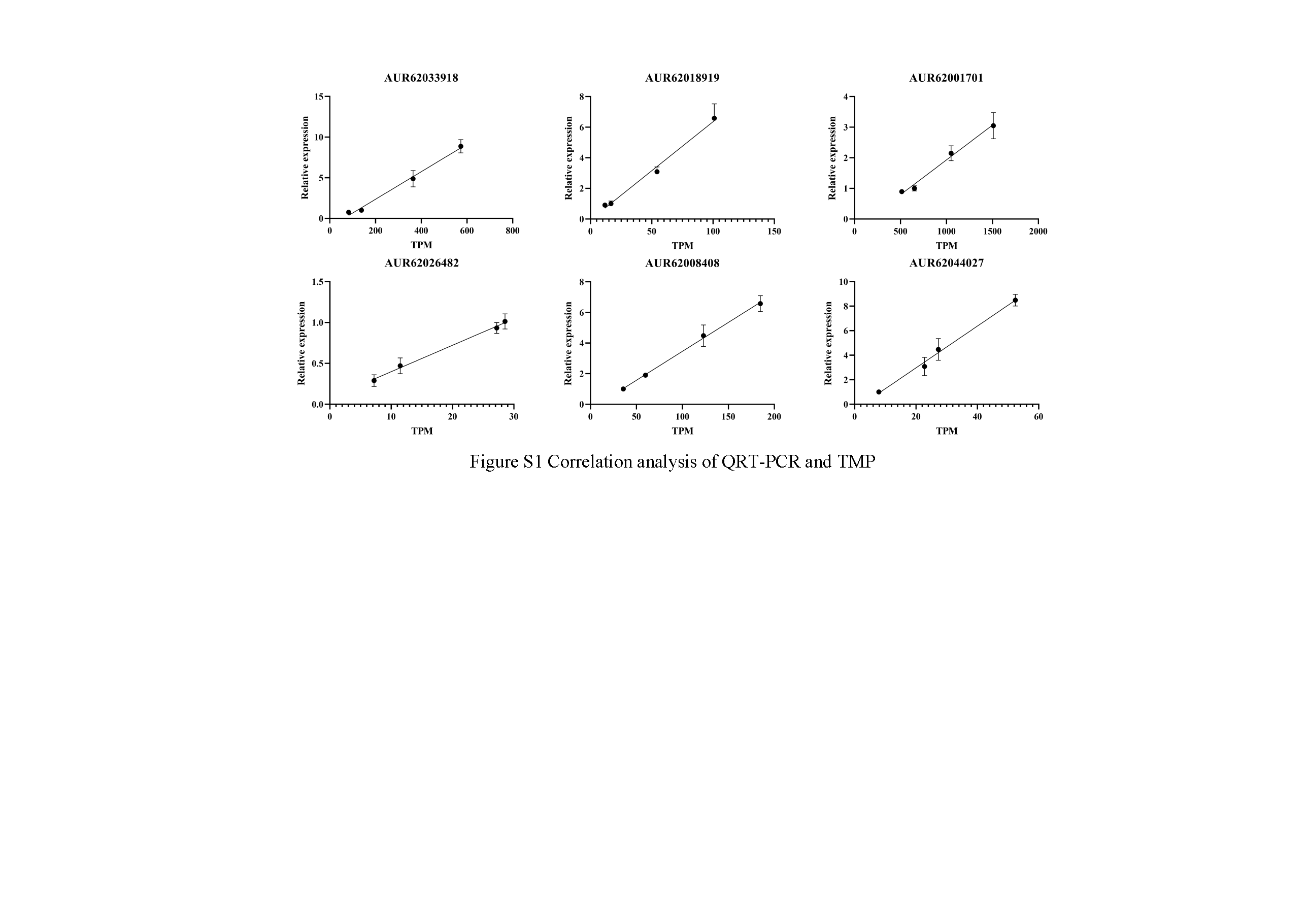

Supplement: Supplementary file 1 [file biology-14-00416-s001.zip › Supplementary(Figure+Table)/Figure S1.tif]

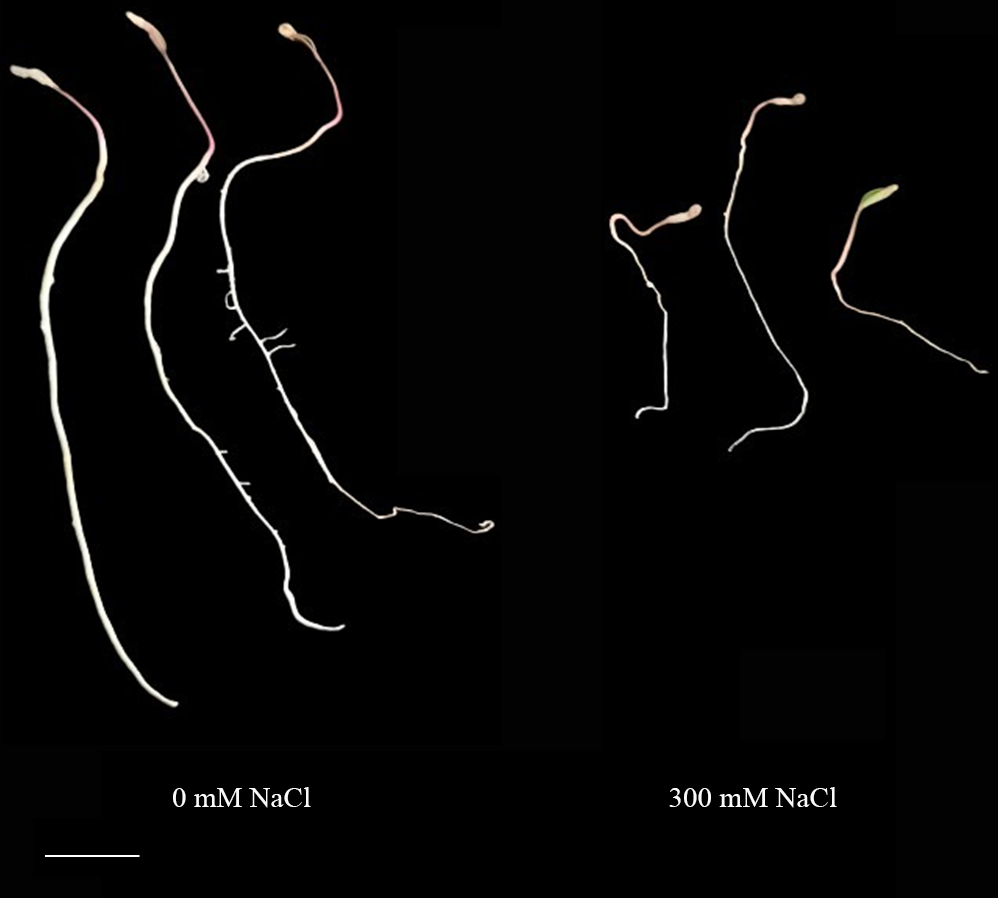

Supplement: Supplementary file 1 [file biology-14-00416-s001.zip › Supplementary(Figure+Table)/Figure S2.tif]

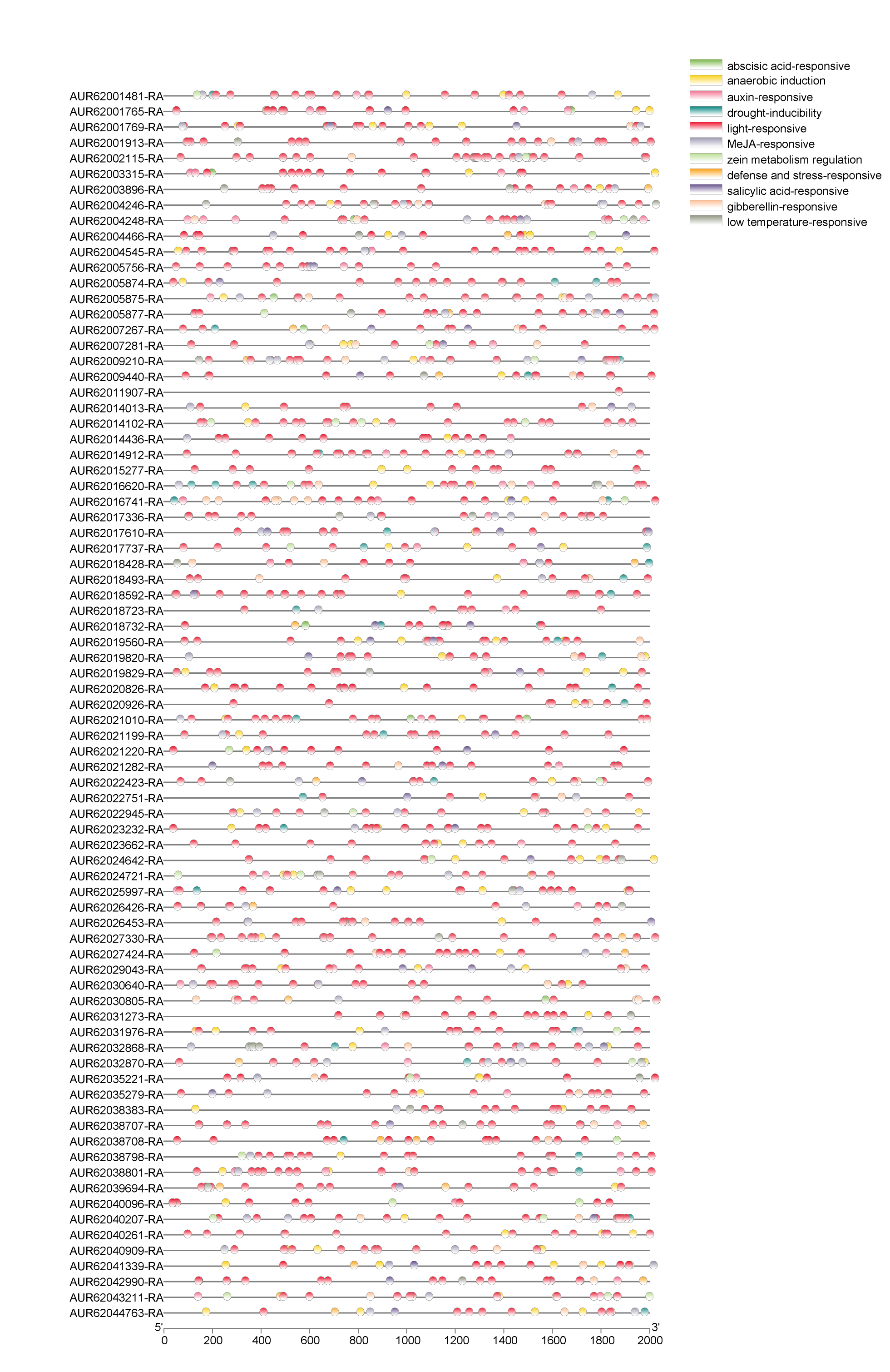

Supplement: Supplementary file 1 [file biology-14-00416-s001.zip › Supplementary(Figure+Table)/Figure S3.tif]

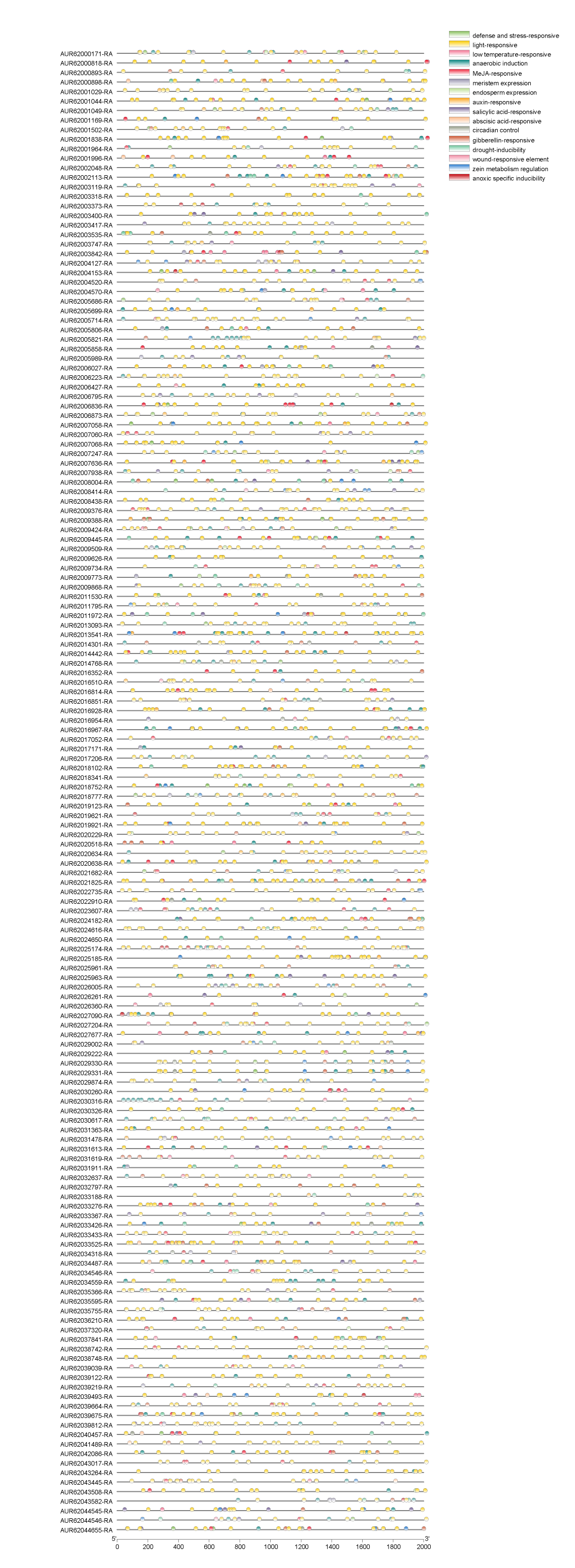

Supplement: Supplementary file 1 [file biology-14-00416-s001.zip › Supplementary(Figure+Table)/Figure S4.tif]
